# Structural insights into RapZ-mediated regulation of bacterial amino-sugar metabolism

**DOI:** 10.1093/nar/gkx732

**Published:** 2017-09-05

**Authors:** Grecia M. Gonzalez, Svetlana Durica-Mitic, Steven W. Hardwick, Martin C. Moncrieffe, Marcus Resch, Piotr Neumann, Ralf Ficner, Boris Görke, Ben F. Luisi

**Affiliations:** 1 Department of Biochemistry, University of Cambridge, Tennis Court Road, Cambridge CB2 1GA, UK; 2 Department of Microbiology, Immunology and Genetics, Max F. Perutz Laboratories, University of Vienna, Vienna Biocenter, 1030 Vienna, Austria; 3 Georg-August University Göttingen, Department of Molecular Structural Biology Justus von Liebig Weg 11, D-37077 Göttingen, Germany

## Abstract

In phylogenetically diverse bacteria, the conserved protein RapZ plays a central role in RNA-mediated regulation of amino-sugar metabolism. RapZ contributes to the control of glucosamine phosphate biogenesis by selectively presenting the regulatory small RNA GlmZ to the essential ribonuclease RNase E for inactivation. Here, we report the crystal structures of full length *Escherichia coli* RapZ at 3.40 Å and 3.25 Å, and its isolated C-terminal domain at 1.17 Å resolution. The structural data confirm that the N-terminal domain of RapZ possesses a kinase fold, whereas the C-terminal domain bears closest homology to a subdomain of 6-phosphofructokinase, an important enzyme in the glycolytic pathway. RapZ self-associates into a domain swapped dimer of dimers, and *in vivo* data support the importance of quaternary structure in RNA-mediated regulation of target gene expression. Based on biochemical, structural and genetic data, we suggest a mechanism for binding and presentation by RapZ of GlmZ and the closely related decoy sRNA, GlmY. We discuss a scenario for the molecular evolution of RapZ through re-purpose of enzyme components from central metabolism.

## INTRODUCTION

Bacterial small regulatory RNAs (sRNAs) are labile components of circuits that dynamically regulate key physiological processes such as metabolic pathways (1–3). In *Escherichia coli*, one metabolic route that is closely regulated through RNA is the biogenesis of glucosamine, an early and essential precursor in the synthesis of the peptidoglycans that are required for the coordinated execution of cell division (4). In that regulatory network, the sRNA GlmZ stimulates translation of the enzyme glucosamine-6-phosphate synthase (GlmS) that acts at the first step of the pathway. Facilitated by the RNA chaperone Hfq, GlmZ base-pairs to an anti-Shine-Dalgarno sequence of the mRNA encoding the synthase (*glmS*) to promote both translation and stability of the transcript (5–6).

**Figure 1.**
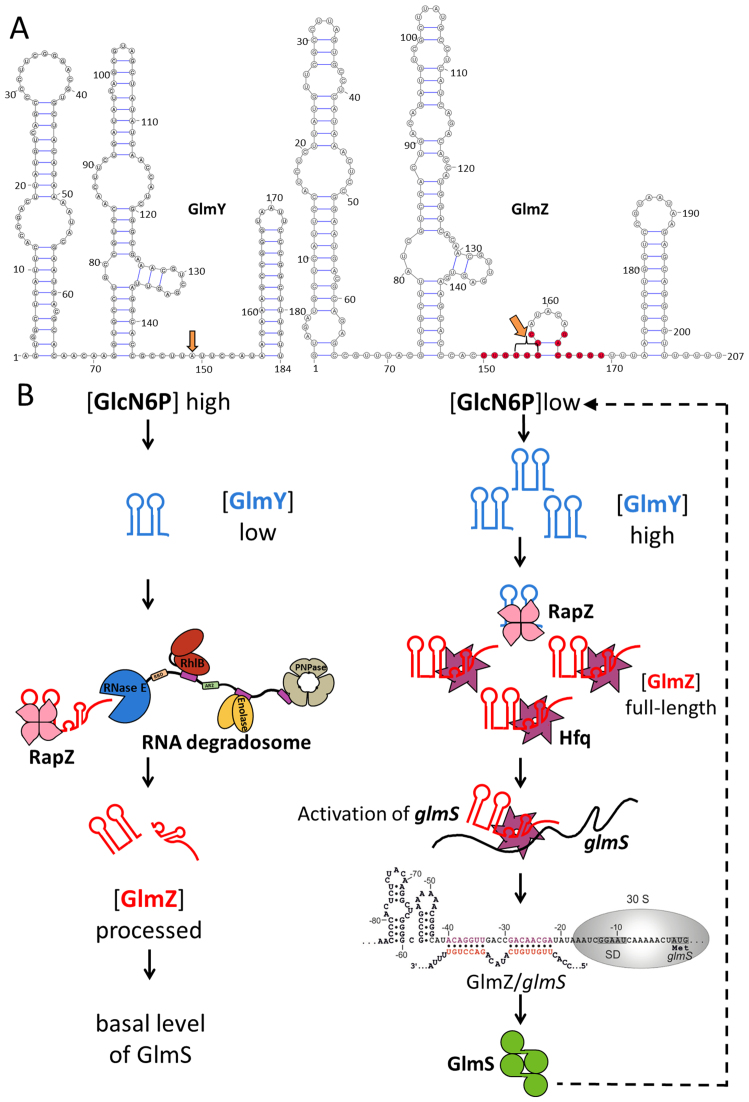
The role of GlmY/GlmZ sRNAs in regulating glucosamine-6-phosphate (GlcN6P) levels in *Escherichia coli*. (**A**) Predicted secondary structures of the sRNAs GlmY and GlmZ (8). The sites that are processed to generate the mature forms of these sRNAs are indicated by orange arrows. GlmZ nucleotides involved in base-pairing with the *glmS* transcript are labelled in red. (**B**) Model for the control of the GlmY/GlmZ cascade by RapZ. When GlcN6P concentrations are high in the cell, GlmY is present in low amounts. Under these conditions, RapZ is free to bind GlmZ and facilitate processing by RNase E, thereby inactivating GlmZ and blocking GlmS synthesis. When GlcN6P levels decrease, processed GlmY accumulates and binds and sequesters RapZ. As a result, GlmZ is free to base-pair with *glmS* in an Hfq-dependent manner and activate synthesis of GlmS, which catalyses the generation of GlcN6P. Figure adapted from Göpel *et al.* (8).

The purely stimulatory activity of GlmZ is counteracted by the ribonuclease RNase E, which cleaves the sRNA into a non-functional shorter variant (of 153/155 nt) lacking the *glmS* interaction site (5–7). The processing event is facilitated by the protein RapZ (formerly named YhbJ), which forms a binary complex with the sRNA for delivery and presentation to RNase E (8). A closely related RNA, GlmY acts as a decoy that is also bound by RapZ but in a manner that occludes GlmZ binding and therefore spares that sRNA from processing and inactivation by RNase E (8–10) (Figure 1). Unlike GlmZ, RapZ-bound GlmY is not a substrate for RNase E (7). Because the levels of GlmY increase when the intracellular concentration of GlcN6P decrease, the resulting inhibition of GlmZ processing indirectly leads to activation of *glmS* (8,10). Contributing to the robustness of the controlling network, GlmY binding to RapZ becomes less favoured with increasing cellular GlcN6P levels (8). The sRNAs GlmZ and GlmY share a high degree of sequence identity (63%) and similar predicted three-dimensional structure, so their dramatically different fates when associated with RapZ present an intriguing puzzle in molecular recognition (7).

RapZ is highly conserved in both Gram-positive and Gram-negative bacteria, implying a crucial role in bacterial physiology (11). The high degree of selectivity *in vivo* for GlmY and GlmZ by *E. coli* RapZ, as indicated by RNA sequencing from co-immunoprecipitates (8), suggests that RapZ might play a highly specialized role. Specificity may be a distinguishing feature of RapZ, as few sRNA-binding proteins have been identified in bacteria thus far that have comparable selectivity (12–14). The target preference of RapZ shares some analogy with the observed specificity of specialized protein cofactors of RNA-mediated regulation in eukaryotes that facilitate targeted processing and degradation of certain microRNAs, which are the counterparts of bacterial sRNAs (15–17).

Currently, there is no structural information available for RapZ that might illuminate the origins of its specificity and functional roles in delivering targets to RNase E. The protein is ∼30 kDa and contains an N-terminal kinase-like domain bearing Walker A and B motifs, which in the *Bacillus subtilis* homologue allows ATP/GTP binding and has been proposed to phosphorylate unknown target proteins (18). Earlier studies suggested that RapZ can interact directly with RNase E, but the determinants of such interaction remain unknown (8). To understand how RapZ functions in complex regulatory processes, we have determined the three-dimensional structure of the *E. coli* protein using X-ray crystallography. We describe the salient structural features of RapZ and their implications for *in vivo* function. Together with functional data presented here and from previous studies, we propose a model for the recognition and presentation of cognate RNAs by this specialized but functionally critical riboregulatory protein.

## MATERIALS AND METHODS

### Growth conditions, plasmids and strains

*Escherichia coli* was routinely grown in Luria Broth (LB) at 37°C under agitation (165 rpm). When required, antibiotics were added to the medium at the following concentrations: ampicillin 100 μg/ml, chloramphenicol 15 μg/ml, kanamycin 30 μg/ml and spectinomycin 50 μg/ml. Expression of genes controlled by *P_Ara_* promoter was induced by the addition of 0.2% L-arabinose and repressed by 0.1% glucose. Established alleles were moved to different strains by general transduction using phage T4GT7 (19). Further details of strain and plasmid construction can be found in the supplementary materials (Supplementary Tables S1 and 2).

### Strep-tag RapZ and RapZ-CTD expression and purification

*Escherichia coli* RapZ and RapZ-CTD were prepared using modifications of the procedure reported earlier (20). The RapZ constructs were expressed in *Escherichia coli* Rosetta (Invitrogen) cells in LB media supplemented with 1% wt/v glucose, 30 μg/ml chloramphenicol, and 100 μg/ml carbenicillin. Cultures were incubated at 37°C, 220 rpm, until the turbidity of the culture reached OD_600_∼0.8, at which point the flasks were placed for 10 min in an ice bath. Expression was induced with 1 mM IPTG (isopropyl β-D-1-thiogalactopyranoside) at 18°C for 1 h for RapZ or 2 hr for RapZ-CTD. Cells were harvested by centrifugation at 4200 × *g* for 20 min at 4°C, and the pellet was then resuspended in lysis buffer (50 mM Tris, pH 8.5, 500 mM KCl, 1 mM ethylenediaminetetraacetic acid (EDTA), 5 mM beta-mercaptoethanol, Protease Inhibitor Cocktail Tablet (Roche) and passed three times through an Emulsiflex-05 cell disruptor (1000–1500 bar; Avestin). The lysate was clarified by centrifugation at 37 500 × *g* for 30 min at 4°C before being passed through a 0.45 μm filter (Millipore) and loaded on a Strep Trap HP column (GE Healthcare) equilibrated with Strep buffer A (50 mM Tris, pH 8.5, 500 mM KCl, 1 mM EDTA, 5 mM 2-mercaptoethanol). Protein was eluted with Strep buffer B (50 mM Tris, pH 8.5, 500 mM KCl, 1 mM EDTA, 5 mM beta-mercaptoethanol, 2.5 mM desthiobiotin) and analysed by sodium dodecyl sulphate-polyacrylamide gel electrophoresis (SDS-PAGE). The Strep-tag was cleaved by Tobacco Etch Virus (TEV) protease for 1 h at room temperature.

For full length RapZ, fractions containing RapZ were pooled and loaded onto a Heparin HiTrap HP column equilibrated with Heparin buffer A (50 mM Tris, pH 8.5, 200 mM KCl, 1 mM TCEP (tris(2-carboxyethyl)phosphine)) and the protein eluted with an isocratic gradient of Heparin buffer B (50 mM Tris, pH 8.5, 1 M KCl, 1 mM TCEP). For RapZ-CTD, fractions were further purified using a MonoS (GE Healthcare) column equilibrated with MonoS buffer A (50 mM Tris, pH 8.5, 100 mM KCl, 1 mM TCEP). Protein was eluted with an isocratic gradient of MonoS buffer B (50 mM Tris, pH 7.5, 1 M KCl, 1 mM TCEP). For both proteins, fractions enriched with RapZ were pooled, concentrated with an Amicon Ultra 30 000 MWCO concentrator (Millipore) and loaded onto a Sephadex 200 Increase gel filtration column (GE Healthcare) equilibrated with buffer containing 50 mM Tris pH 7.5, 100 mM NaCl, and 50 mM KCl, and 2 mM dithiothreitol (DTT). The purest fractions of RapZ were pooled and concentrations determined spectrophotometrically using a molar absorptivity value at 280 nm of 22920 M^−1^cm^−1^ for RapZ or 19940 M^−1^cm^−1^ for RapZ-CTD. Samples were either used immediately for assays or supplemented with 10% v/v glycerol, flash frozen, and stored at −80°C.

### Un-tagged RapZ expression and purification

*Escherichia coli* CSH50 (R1279) cells were transformed with plasmid pFDX4324 expressing *E. coli* RapZ from a constitutive promoter and grown in 2 × YT media at 37°C until an OD_600_ of 0.9 to 1.4 was reached. The harvested cell pellet was resuspended in 50 mM Tris-Cl pH 7.5, 50 mM NaCl, 1 mM TCEP, 5% v/v glycerol, and frozen with liquid nitrogen. The thawed cells were lysed by passage through an Emulsiflex-05 cell disruptor (1000–1500 bar; Avestin). Solid ammonium sulfate was added to 30.8% saturation, and the solution centrifuged at 37 500 × *g* at 5°C for 30 min. Ammonium sulfate was added to the supernatant to 46.2% saturation, spun again, and the pellet dissolved in 50 mM Tris-Cl pH 7.5, 50 mM NaCl, 1 mM TCEP, 5% v/v glycerol. The dissolved pellet was mixed with 1:1 volume of butyl sepharose A buffer (50 mM Tris-Cl pH 7.5, 100 mM NaCl, 50 mM KCl, 2.0 M ammonium sulfate) and applied to butyl sepharose HiTrap HP column, washed with buffer A followed by a gradient 0→60% butyl buffer B (50 mM Tris-Cl pH 8.5, 50 mM NaCl, 10 mM KCl, 0.02% wt/v beta-dodecylmaltoside, 5% v/v glycerol) until baseline stabilized, then continue gradient to 100% B. Fractions enriched in RapZ were pooled, diluted 1:1 with water and applied to Resource Q equilibrated with buffer A (50 mM Tris-Cl pH 8.5, 50 mM NaCl, 10 mM KCl) and eluted with a linear gradient 0→ 20% B (A + 2 M NaCl). Fractions enriched in RapZ were pooled, diluted 1:1 with chilled distilled water, applied to MonoQ and eluted with a gradient 12 to12.7% ResourceQ buffer A to B. Enriched fractions were concentrated with a 10 kDa MWCO centrifugal concentrator, and applied to a S200 increase 10/300 GL equilibrated with Resource Q buffer A.

### Binding kinetic measurements

Kinetic measurements using bio-layer interferometry (BLI) were performed using an Octet RED96 with Streptavidin sensors (ForteBio, UK) on 96-well plates. The experiment was performed in binding buffer (25 mM Tris, pH 7.5, 50 mM NaCl, 50 mM KCl, 1 mM MgCl_2_, 1 mM DTT), which was also used to prepare all dilutions, for dissociation and neutralization. Full-length GlmZ sRNA, processed GlmZ and processed GlmY were *in vitro* transcribed from a polymerase chain reaction template and labelled with biotin using a five-fold excess of GMP-biotin (TriLink Biotechnologies, San Diego CA, USA) over GTP. Each 5′ biotin labelled RNA was immobilized on the biosensor, which was subsequently submerged into a 10 μM solution of maltose binding protein labelled with biotin. The binding of full length RapZ and RapZ-CTD were assayed at 0, 7.5, 15, 25, 50, 75, 125 and 250 nM protein over 400 s. The dissociation was monitored over 300 s and was followed by regeneration of the sensors using 1 M MgCl_2_. Another set of tips was saturated with MBP-biotin and the measurements were then repeated for all protein–RNA concentration series. The data were fitted with Data Analysis software (ForteBio) with a 1:1 binding model. The plots were prepared with ProFit (Quantum Soft, Switzerland) using the following equation for response fit:

\begin{equation*} {\rm{Y}} = {\rm{Rm}}\; \times \; {\rm{X^n}}/({\rm{K_{d}^{n}}} + {\rm{X^n}}), \end{equation*}

where Y is the observed binding, X is the molar concentration of the ligand, Rm is the maximum specific binding, and n is the Hill’s coefficient.

### Size-exclusion chromatography multi-angle light scattering (SEC-MALS)

Protein solution was loaded onto a Superdex 200 10/300 GL (GE Healthcare) at a concentration of 30 μM and a flow rate of 0.5 ml min^−1^. For RapZ-CTD, the column was pre-equilibrated with 25 mM Tris pH 7.5, 200 mM KCl, 1 mM TCEP. Multi-angle light scattering analysis was performed continuously on the column eluate at 25°C using DAWN HELEOS and Optilab T-rEX triple-angle light-scattering detectors (Wyatt Technology Corp.). The data were processed with ASTRA 6.1.1.17 software (Wyatt Technology Corp.).

### Sedimentation velocity analytical ultracentrifugation (SV-AUC)

Sedimentation velocity experiments were performed using a Beckman Optima XL-I analytical ultracentrifuge. Sample concentrations were 0.5–1 mg ml ^−1^ and data were collected at a wavelength of 280 nm. The samples were spun at 20°C, 50 000 rpm using an An60Ti rotor (Beckman) for 3–4 h. Partial specific volumes were estimated using the program Sednterp and the data were fit and modelled using Sedfit (21).

### Crystallographic analysis of RapZ-CTD

Crystals of RapZ-CTD were obtained using the vapor diffusion method with a well solution containing 100 mM HEPES, pH 7.0 and 1 M tri-sodium citrate (condition G12 from ProPlex Screen, Molecular Dimensions) at room temperature over the course of 3 days. Malonate was found to have a favourable effect on the size and stability of the crystals. Diffracting crystals were grown using the hanging drop vapour diffusion method where 2 μl of RapZ-CTD at 4 mg/ml in 20 mM Tris, pH 7.5, 100 mM NaCl, 50 mM KCl, 1 mM DTT was mixed with 2 μl of a reservoir of 100 mM HEPES, pH 7.0 and 1 M tri-sodium citrate or 1 M malonate. Crystals were cryoprotected by direct addition of ethylene glycol diluted in reservoir buffer into the crystal drop. Heavy metal derivatives were prepared by soaking crystals in different heavy metal solutions at concentrations ranging 1–10 mM and added directly to the drop for between 10 s and 3 min.

A native dataset at 1.17 Å resolution, and two single wavelength anomalous Au and Pt derivative datasets at 1.69 Å and 1.79 Å resolution, respectively, were collected on beam line I03 at Diamond Light Source (Didcot, Oxfordshire). The heavy metal substructure was determined using HYSS and initial phases, density modification, and model building was performed by PHASER and RESOLVE as implemented automatically by PHENIX (22). The asymmetric unit consisted of a single copy of RapZ-CTD. Manual rebuilding was performed using COOT (23). Reciprocal space refinement with local non-crystallographic symmetry constraints was performed using PHENIX with the native dataset (22). Difference density that was too large for ordered water was modelled as malonate, which was present in the crystallization condition. The final model has an R_work_ of 0.148 and an R_free_ of 0.168 with residues in the Ramachandran plot in 97.6% preferred regions and none in the disallowed and restricted regions. Crystallographic data statistics are summarized in Table 1.

**Table 1.** Crystallographic data collection and refinement statistics for RapZ-CTD

	Native (5O5S)	Pt derivative	Au derivative
Space group	P6_5_22	P6_5_22	P6_5_22
Cell dimensions (Å)	a = 61.72, b = 61.72, c = 124.53	a = 61.94, b = 61.94, c = 124.49	a = 61.78, b = 61.78, c = 124.57
No. of observations	428987	265911	415316
No. of unique observations	46875	14045	16515
Resolution (Å)	1.17 (62.29)	1.79 (53.64)	1.69 (62.28)
Completeness (%)	99.57	99.62	99.47
Anomalous completeness (%)		99.42	99.14
Multiplicity	9.2 (4.4)	18.9 (11)	25.1 (10)
Anomalous multiplicity		10.3 (5.7)	13.5 (5.3)
CC_1/2_	0.999 (0.599)	0.999 (0.767)	0.999 (0.486)
Rmerge	0.085 (0.888)	0.107 (1.651)	0.115 (1.669)
I/SigmaI	9.5 (1.2)	13.1 (1.3)	15.4 (1.3)
R factor	0.154		
R free	0.176		
RMSD bond lengths (Å)	0.012		
RMSD bond angles (°)	1.21		
Ramachandran Favored (%)	96.58		
Ramachandran Preferred (%)	3.42		
Ramachandran Outliers (%)	0		

*Highest resolution shell is shown in parenthesis.

### Structure solution and refinement for full-length RapZ

The structure of RapZ-CTD was used as the starting model for molecular replacement performed with PHASER (22). Four subunits of RapZ-CTD were placed within the asymmetric unit of both the P32 and P3221 datasets. In order to overcome the difficulties in manual model rebuilding based on the low resolution electron density map, the HHPRED server was used to search the PDB for closely related homologous structures. The best scoring templates were subjected to energy and density-guided refinement in Rosetta utilizing constraints from an averaged electron density maps calculated with PARROT from the CCP4 suite of crystallographic programs (24). Resulting models were scored using the internal Rosetta score function and merged into an almost complete model of NTD. The missing three copies of NTD were placed manually and the full model was refined using REFMAC5 with TLS refinement, NCS and Jelly Body restraints (25). The model was manually rebuilt in Coot (23) and verified against simulated annealing omit maps. The final structure is presented in Figure 4. Overall, unambiguous electron density throughout most of each subunit chain allowed residues 1–281 to be modelled. Areas for which electron density could not be observed include the linker regions connecting the NTD and CTD (residues 153–159) of Chains C and D and a disordered loop (residues 99–112) on Chains B and A. The same loop is, however, modelled in Chains C and D where unambiguous density allowed it to be built. Crystallographic data statistics are summarized in Table 2.

**Table 2.** Crystallographic data collection and refinement statistics of full length RapZ

	RapZ–P3221 (5O5Q)	RapZ–P32 (5O5O)
Beamline	BESSY BL14.1	ESRF, ID23–2
Wavelength (Å)	0.91841	0.87260
Space group	P3_2_21	P3_2_
Cell dimensions (Å)	a = 91.54, b = 91.54, c = 352.55	a = 92.65, b = 92.65, c = 156.61
Unique reflections	26027	19961
Resolution (Å)	44.34–3.26	46.33 - 3.40
Completeness (%)	97.6	99.7
Multiplicity	4.7 (4.8)	3.5 (3.5)
CC_1/2_	0.998 (0.768)	0.995 (0.575)
Rmerge	0.086 (0.762)	0.123 (0.822)
I/σ(I)	13.37 (2.56)	11.2 (1.8)
R factor	0.2176	0.1973
R free	0.2729	0.2663
RMSD bond lengths (Å)	0.004	0.011
RMSD bond angles (°)	0.7	1.3
Ramachandran favoured (%)	95.09	97.16
Ramachandran preferred (%)	4.33	2.37
Ramachandran outliers (%)	0.58	0.47
MolProbity score	1.86	1.75
Clashscore	9.87	12.09

*Highest resolution shell is shown in parenthesis.

### Bacterial two-hybrid (BACTH) assays

The bacterial two-hybrid (BACTH) system is based on reconstitution of adenylate cyclase activity in *E. coli* strain BTH101, which lacks a functional endogenous *cyaA* gene (26). Reconstitution occurs through the interaction of proteins fused to the T25- and T18-domains of split adenylate cyclase from *Bordetella pertussis*. In this study, full-length RapZ or its truncated forms (RapZ aa 1–152 and RapZ aa 153–284), as well as their mutant variants were fused to the T25- and/or T18- domains and tested for their ability to self-interact. Derivatives of plasmids pUT18C and pKT25 encoding the latter RapZ variants were introduced into strain BTH101 and the resulting transformants were grown at 28°C to stationary phase in LB containing 1 mM IPTG and the required antibiotics and the β-galactosidase activities were determined.

### β-galactosidase assays

β-galactosidase activity assays were determined as previously described using cells harvested in the exponential growth phase (OD_600_ = 0.5–0.8) if not otherwise indicated (27). All values represent the average of at least three measurements using independent cultures.

### *In vitro* transcription and labelling of small RNAs

Full-length GlmY was internally labelled by α-^32^P-UTP using T7 polymerase, as described previously (8). Extraction of radiolabelled RNA from the gel was performed as described previously (28).

### Electrophoretic mobility shift assays (EMSA)

Electrophoretic mobility shift assays (EMSA) experiments were performed as described earlier (7). Briefly, 4 nM radiolabelled GlmY was mixed with 1 μg yeast tRNA (Ambion), heat-denatured and mixed with various amounts of RapZ and its variants in 1× binding buffer (10 mM Tris pH = 7.0, 100mM KCl, 10mM MgCl_2_) in a reaction volume of 10 μl. After incubating for 30 min at 30°C, samples were mixed with 2 μl of 5 × native loading buffer (50% v/v glycerol, 0.5 × TBE, 0.2% bromophenol blue) and separated by electrophoresis at 4°C on a native gel (6% acrylamide, 1 × TBE) for 2–3 h at 300V using 0.5 × TBE as running buffer. Dried gels were analysed by phosphoimaging.

Strep-tagged proteins used in the EMSA experiments were overproduced from plasmids in strain Z864, which lacks endogenous *rapZ, glmY* and *glmZ*. Cultures were grown in 100 ml LB-ampicillin to an OD_600_ of 0.5–0.8 and induced with 1 mM IPTG. One hour after induction, cells were harvested by centrifugation and pellets were re-suspended in Buffer W (100 mM Tris-Cl, 300 mM NaCl and 1 mM EDTA, pH 8.0). Cells were lysed either by sonication or French pressure cell at 18 000 psi and lysates were cleared by centrifugation. Supernatants were subsequently loaded onto 0.2 ml StrepTactin suspension (IBA) equilibrated with Buffer W in 10 ml poly-prep chromatography columns (Bio-Rad). After four washes with 10 × column bed volume of Buffer W, proteins were eluted using 3 × 0.1 ml Buffer E (100 mM Tris-Cl pH 8.0, 150 mM NaCl and 1 mM EDTA, 2.5 mM desthiobiotin). All fractions containing pure protein were pooled and dialysed twice for 20–24 h in dialysis buffer (10 mM Tris-Cl pH 7.0, 100 mM KCl, 10 mM MgCl_2_, 2 mM β-mercaptoethanol). Five percent v/v glycerol was added to the proteins for storage at −80°C.

### Protein/RNA copurification

Copurification experiments were carried out as previously described (8) with minor alterations. Strain Z903 (*ΔrapZ*) overproducing the Strep-tagged protein of interest was grown in 500 ml LB, harvested and cell pellets were resuspended in 20 ml buffer W (100 mM Tris-Cl pH 8.0, 150 mM NaCl and 1 mM EDTA) and lysed in a French pressure cell at 18 000 psi. Protein purification via StrepTactin affinity chromatography was performed as described above but 5 × higher volumes of the various buffers were used. A total of 250 μl of the second elution fraction were subjected to phenol:CHCl_3_:isoamyl alcohol (25:24:1) extraction and RNA was subsequently precipitated using ethanol:4 M LiCl (30:1) at −20°C overnight and dissolved in 20 μl RNase free water. For northern analysis, the volumes of the RNA samples were adjusted to the concentrations of the purified proteins. A maximum of 3 μl was loaded.

### Extraction of total RNA and northern blotting

Total RNA was extracted using the ReliaPrep RNA Cell Miniprep System (Promega). Three micrograms of total RNA and varying amounts of RNA extracted from protein eluates were analysed by northern blotting, as was previously performed (10). RNA was hybridized with a Digoxigenin-labelled GlmY-specific probe that was generated using the DIG RNA Labeling kit and detected using the CDP* detection system (Roche Diagnostics) according to the manufacturer’s instructions.

### RNase E (1-529) expression and purification, and use in RNA degradation assays

RNase E (1–529) was prepared as described previously (29). To achieve a final RNA concentration of 20 nM, 1 μl of 2 μM RNA was heat annealed by incubation for 2 min at 50°C in reaction buffer (25 mM Tris-Cl pH 7.5, 50 mM NaCl, 50 mM KCl, 1 mM MgCl_2_, 1 mM DTT, 0.5 U/μl RNaseOUT (Invitrogen) in a volume of 10 μl. The solution was allowed to cool to room temperature over 3 min. Subsequently, 1 μl of the renatured RNA solution was added to an 8 μl volume containing 60 nM RapZ, and the solution was incubated at 30°C for 15 min. Cleavage was initiated by the addition of 1 μl of reaction buffer containing the catalytic domain of RNase E (final concentration 15 nM), followed by incubation for the indicated amount of time at 30°C. Time course reactions were stopped after 1, 2, 3, 5, 15, 30 min by the addition of Proteinase K in 2× PK Buffer (200 mM Tris-Cl pH 7.5, 25 mM EDTA, 300 mM NaCl, 2% wt/v sodium dodecylsulfate), and the samples were incubated for 15 min at 50°C. RNA loading buffer (2×) was added, and the samples were separated on a denaturing 10% polyacrylamide gel. RNA was visualized by SYBRGold staining and UV imaged using GeneSnap software.

## RESULTS

### The crystal structure of the C-terminal domain of RapZ

**Figure 2.**
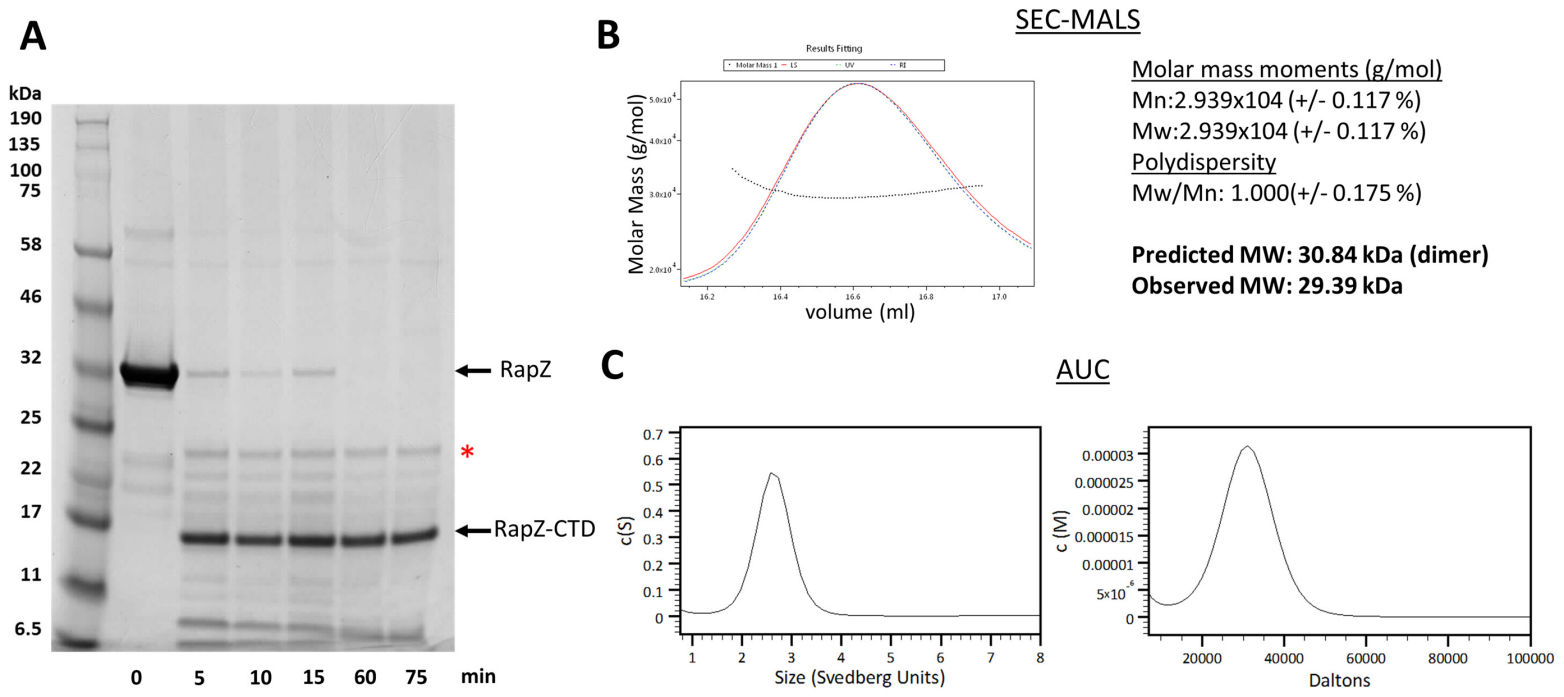
A protease-resistant RapZ C-terminal region forms a structurally autonomous domain that homodimerises. (**A**) Limited proteolysis reveals a truncated species resistant to digestion. Full-length RapZ (1 mg/ml) was digested by trypsin (0.02 mg/ml) over the course of 75 min at 37°C, revealing a truncated species resistant to digestion spanning residues 154–284, as confirmed by mass spectrometry (data not shown). This residue range corresponds to the C-terminal RNA-binding domain of RapZ. The red asterisk denotes a band corresponding to trypsin used for digestion. (**B**) SEC-MALS analysis (left panel) reveals that RapZ-CTD elutes as a single peak on the chromatogram. Analysis of the peak fractions is summarized in the right panel. (**C**) AUC analysis in the sedimentation velocity mode of RapZ-CTD. The left and right panels show distributions for sedimentation coefficient (c(S)) and molecular mass (c(M)), respectively.

Diffracting crystals of full-length RapZ have been previously obtained (20), but due to lack of experimental phases or a suitable model for molecular replacement the crystal structure has remained unsolved. We endeavoured to solve this crystal structure piece-wise using models for the stable domains. Using limited proteolysis, the C-terminal half of RapZ (encompassing residues 154–284) was identified as a domain that could withstand digestion (Figure 2A). The corresponding domain, RapZ-CTD, was overproduced and purified. Analysis by SEC-MALS (size-exclusion chromatography with multi-angle light scattering) and AUC (analytical ultracentrifugation) indicate that in isolation this protein domain is a homodimer (Figure 2B and C).

Well-ordered crystals were obtained of the purified RapZ-CTD, and a native dataset from a single crystal was collected at 1.17 Å resolution. Single wavelength anomalous datasets from two Au and Pt derivative crystals were collected at 1.69 Å and 1.79 Å resolution, respectively. Experimental phases were obtained by isomorphous differences combined with anomalous signals using the PHENIX programs (22). Overall, the electron density was readily interpretable and allowed residues 156–281 to be modelled. Complete crystallographic statistics are summarized in Table 1.

**Figure 3.**
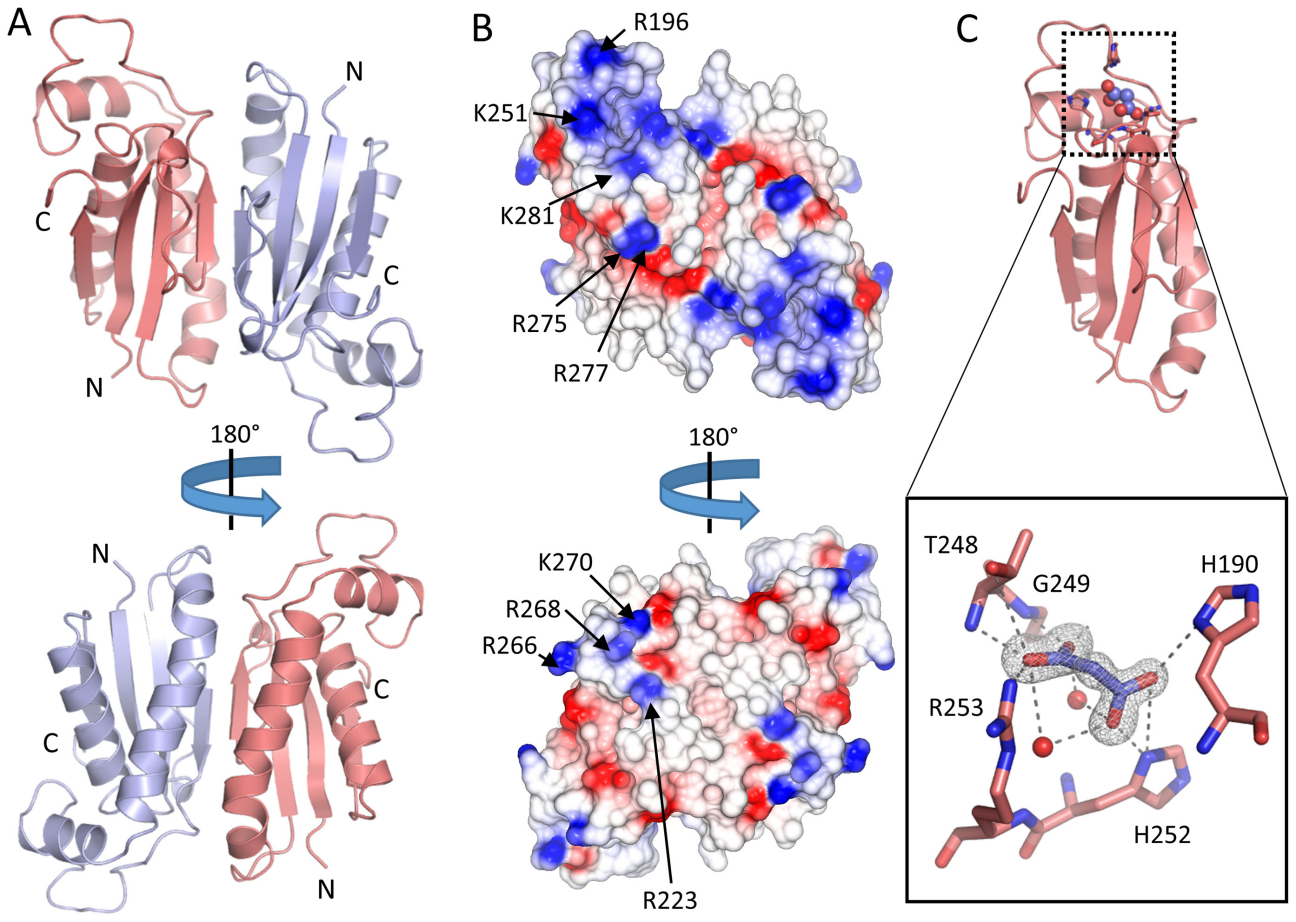
The X-ray crystal structure of the C-terminal domain of RapZ. (**A**) The X-ray crystal structure of RapZ-CTD in two orientations shown as cartoon representation. The two RapZ-CTD protomers are coloured blue and red. (**B**) The RapZ-CTD dimer shown as an electrostatic surface representation. Two views as in (A), with electropositive regions coloured in blue and electronegative regions coloured in red. Electropositive residues previously predicted to be involved in RNA binding (8) are labelled on one protomer of the RapZ-CTD dimer. (**C**) The malonate binding pocket of the RapZ-CTD. Top: a protomer of RapZ-CTD shown in the same orientation as 3A, as a red cartoon with residues contacting the bound malonate shown as sticks. Bottom: a close up view of a putative ligand-binding pocket that is occupied by malonate. Malonate is shown as blue sticks, with a F_o_-F_c_ omit map for the malonate shown as grey mesh contoured at 3 σ. Residues coordinating the malonate are shown as red sticks, and water molecules are shown as red spheres. Hydrogen bonds are represented by grey dashed lines.

The structure of RapZ-CTD is presented in Figure 3. The protein forms a crystallographic dimer with a symmetry-related copy (Figure 3A), burying 1051.5 Å^2^ of solvent accessible surface area as determined by PISA analysis (30). This self-interacting interface is likely to account for the stable homodimer observed in solution (Figure 2B and C). The electrostatic surface of the RapZ-CTD dimer is shown in the Figure 3B and indicates basic patches that may play a role in RNA binding. Of note are a cluster of basic residues around an extended loop on the surface of RapZ-CTD, including arginine 196 and lysines 251 and 281. On the opposite face of the dimer is a small electropositive surface protrusion composed of arginine residues 223, 266, 268 and Lys270. These residues, with the exception of Arg196, have previously been predicted to be involved in RNA binding, and it has been shown that mutation of Lys270 in conjunction with Lys281, Arg282 and Lys283 does indeed abolish RNA binding by RapZ (8). It should be noted that residues 281, 282 and 283 at the C-terminus of RapZ are not resolved in our crystal structure.

During the process of modelling and refinement, clearly defined electron density for a non-protein ligand was observed in a pocket composed of the side chains of residues His190, His252, Arg253 and the backbone of Thr248 and Gly249 (Figure 3C). During optimization of the protein crystals, we found the addition of malonate to be extremely beneficial for crystal growth, and a malonate molecule accounts for this density excellently.

The refined crystal structure of RapZ-CTD was used to search for structural homologues using the DALI server (31). Multiple hits were returned for structures of 6-phosphofructokinase (PFK). The highest scores (z 6.2–6.0 with 3.7 Å rmsd and 13–12% identity) were for a 91 amino acid segment within PFK subunit alpha from rabbit skeletal muscle (PDB code: 3O8L). The overlap corresponds to the domain of PFK that binds fructose-6-phosphate, although the binding pocket is not conserved in RapZ. In comparison to the PFK alpha subunit, the RapZ fold is elaborated with an inserted loop (residues 186–215). The corresponding region from PFK is involved in a dimerization interface, but this contacting surface is on the opposite face from that used to form RapZ-CTD dimers. A structural comparison of RapZ-CTD and PFK is shown in Supplemental Figure S1.

### Crystal structure of full length RapZ reveals a homotetramer with a kinase fold for the N-terminal domain

The refined model of RapZ-CTD was used to solve the crystal structure of the full-length protein by molecular replacement with the two previously collected X-ray diffraction datasets (space groups P32 and P3221) (20). Four subunits of RapZ-CTD could be accommodated within the asymmetric unit of both datasets, and the calculated electron density map provided sufficient definition to manually build secondary structure features for the NTD starting from the CTD-based core of the tetrameric assembly. Overall, unambiguous electron density throughout most of each subunit chain allowed residues 1–281 to be modelled and refined. Areas for which electron density could not be observed include regions connecting the CTD and N-terminal domain (hereafter, NTD) of Chains C and D (residues 153–159) and a loop (residues 99–112) that is disordered in both Chains A and B. The same loop is, however, modelled in Chains C and D where the density was unambiguous. The crystallographic refinement statistics are summarized in Table 2. The overall topology and structural arrangement of RapZ is almost identical within the two distinct crystal forms.

**Figure 4.**
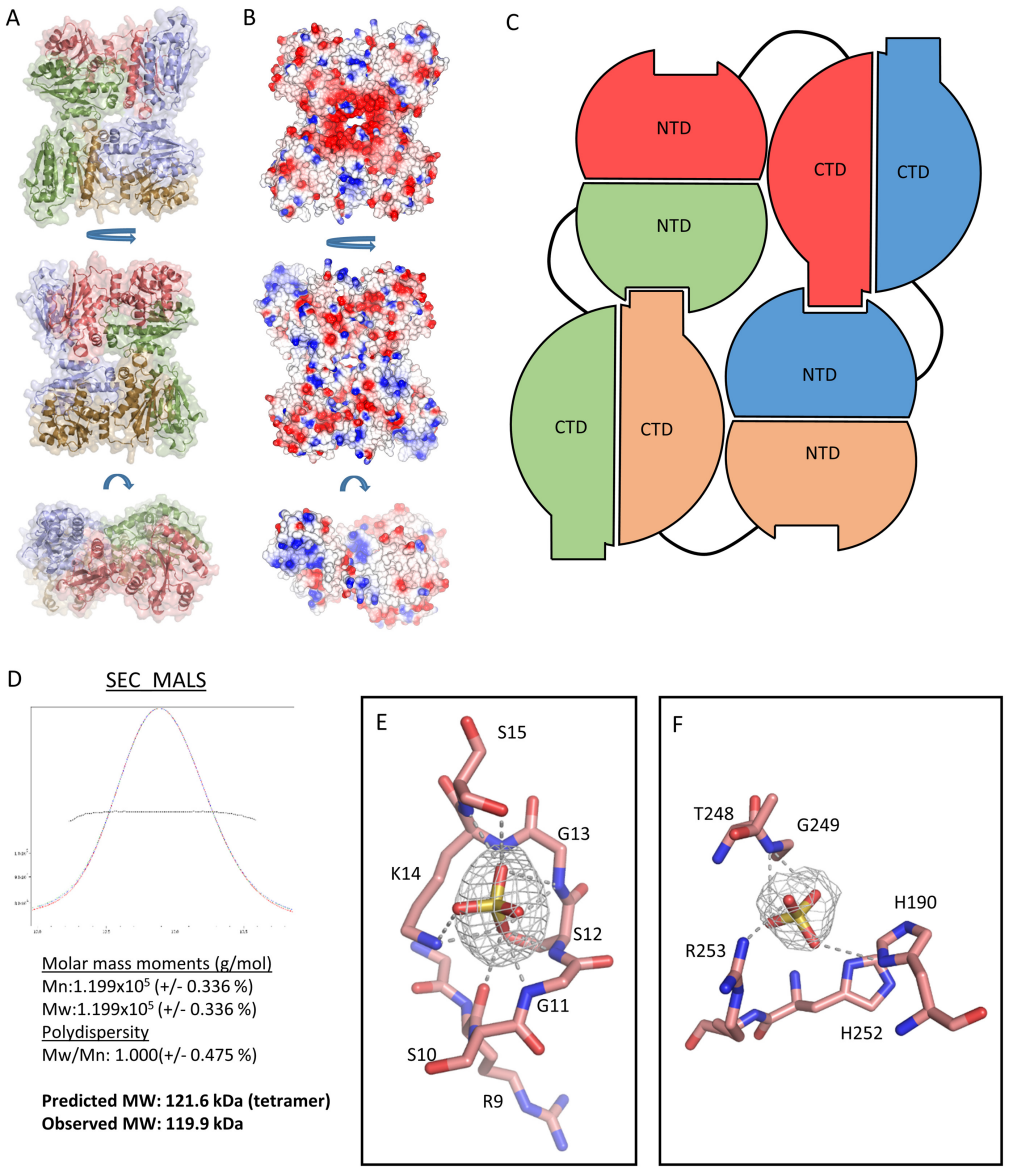
The X-ray crystal structure of RapZ. (**A**) The X-ray crystal structure of RapZ in three orientations in cartoon representation with semi-transparent surface. The RapZ protomers are coloured red, blue, orange and green. (**B**) Electrostatic surface representation of the RapZ tetramer in three orientations as in A. Electropositive regions are coloured blue and electronegative regions are coloured red. (**C**) Diagram representation of the global domain architecture of RapZ. The protomers of RapZ are coloured red, blue, orange and green as in A. The protein assumes a tetrameric assembly in the crystal structure wherein some NTD-CTD contacts are satisfied whereas others are not. It is formally possible that in solution the tetramer is reorganized as a self-closing dimer of dimers that satisfies all the NTD-CTD contacts. (**D**) SEC-MALS analysis (top) reveals that RapZ elutes as a single peak in the chromatogram. Analysis of the peak fractions is summarized in the bottom panel. (**E**) A close up view of the putative Walker A-box in a single protomer of RapZ. Bound sulfate is shown as yellow and red sticks, with a F_o_-F_c_ omit map for the ligand shown as grey mesh contoured at 3 σ. Residues forming the Walker-A box are shown. Hydrogen bonds are represented by grey dashed lines. (**F**) Sulfate modelled bound at the ‘malonate’ binding site in the RapZ-CTD (In the same orientation as Figure 3C). Bound sulfate is shown as yellow and red sticks, with a F_o_-F_c_ omit map for the ligand shown as grey mesh contoured at 3 σ. Residues coordinating the ligand are shown as red sticks. Hydrogen bonds are represented by grey dashed lines.

The crystal structure and electrostatic surface representation of the RapZ tetramer are presented in Figure 4A and B, respectively. The tetrameric assembly is composed of a dimer of dimers in which the NTD and CTD form two homodimeric pairs. The CTD forms tightly packed dimers in a manner that exactly resembles the dimers seen in the RapZ-CTD structure. The NTDs appear to extend from their respective CTDs to form dimers with packing that, similar to the CTD, appears to be driven by interactions along parallel beta strands and adjacent helices between the subunits. SEC-MALS analysis of purified RapZ is supportive of a tetramer in solution (molecular mass estimated 119.9 kDa, versus expected mass of 121.6 kDa; Figure 4D). Interestingly the overall assembly is asymmetric as a consequence of flexibility in the NTD dimers. Moreover, the quaternary structure comprises a domain swapped dimer-of-dimers formed through the interlacing of the subunits. This organization is depicted schematically in Figure 4C.

The domain swapped tetramer as seen in the crystal structure could conceivably allow continuous polymers of RapZ to be formed as on each tetrameric assembly there are unsatisfied interaction sites on both the NTD and the CTD. However, we have seen no evidence of the formation of filament-like structures of RapZ during the purification of this protein. It is possible that in solution the tetramer is reorganized as a self-closing dimer-of-dimers.

The fold of the NTD closely matches that of eukaryotic and archaeal kinases, with greatest similarity identified from the DALI server to an adenylate kinase-related protein from *Sulfolobus solfataricus*. For this homolog (PDB code: 3LW7), the rmsd (root-mean-square deviation) between aligned 131 Ca atoms amounts to 2.6 Å, with a Z-score of 12.5 and 16% sequence identity (Supplementary Figure S2). More locally, the Walker A motif closely matches that found in numerous other proteins, and indeed, putative sulfate ions are observed bound to this motif in each RapZ subunit, coordinated primarily via backbone amides (Figure 4E). The less conserved Walker B motif, however, appears to be disordered and not involved in interactions with the Walker A box.

Additionally, electron density is observed in the pocket where malonate was identified in our RapZ-CTD structure. This density would not accommodate malonate (and indeed malonate was not present in the crystallization condition). Instead, we have modelled a sulfate ion in this position as this was an abundant ion in the crystallization condition (Figure 4F). It is likely that this pocket could bind the phosphate moiety of a ligand.

### *In vivo* analysis confirms RapZ self-interaction

**Figure 5.**
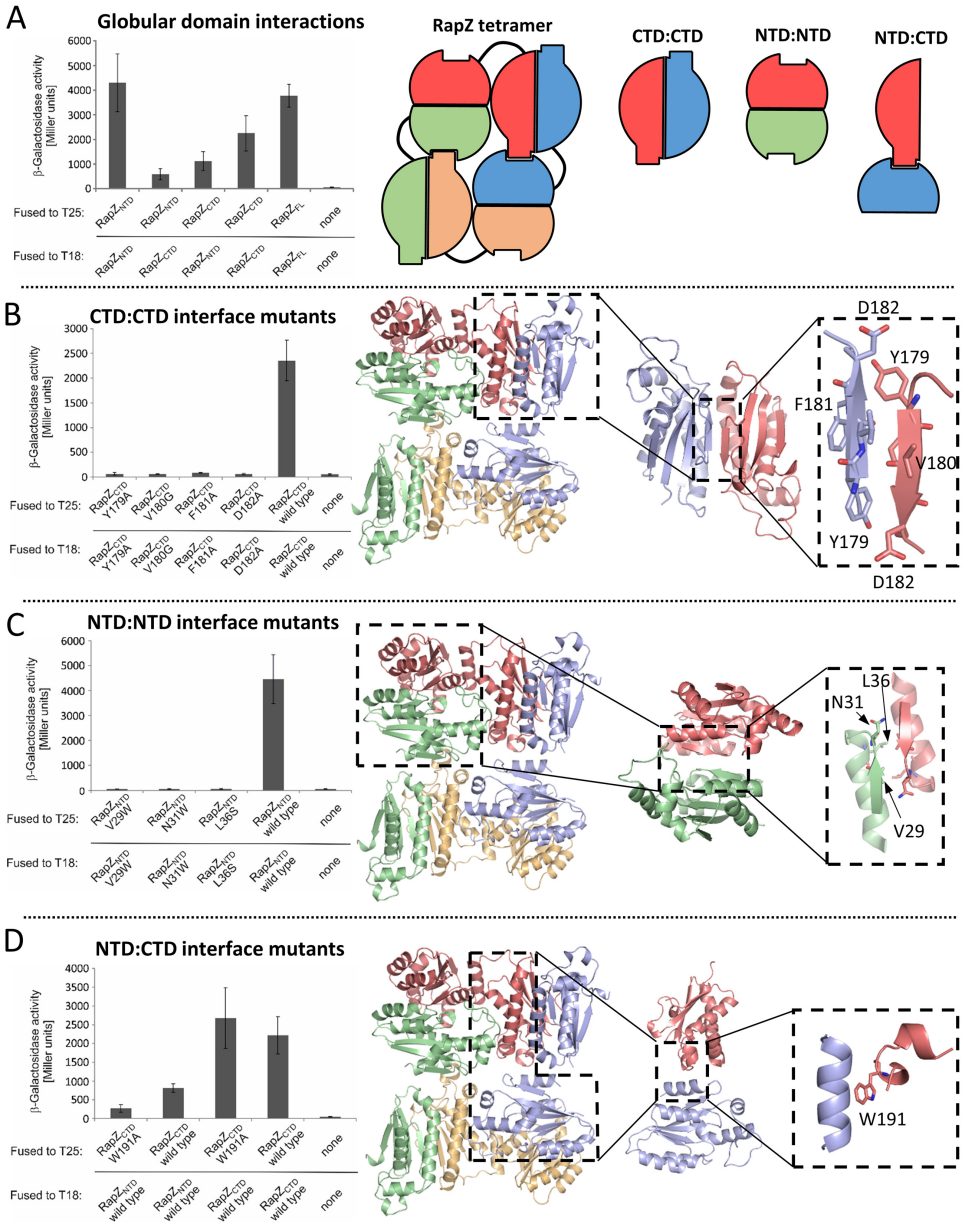
Self-interaction of RapZ domains *in vivo*. (**A**) RapZ NTD and CTD in isolation can form homo-oligomers and they also cross-interact in BACTH assays. Strains carrying plasmids encoding for the T18- and T25- fusion proteins as indicated in the left panel were grown to stationary phase and the β-galactosidase activities were measured. Plasmids encoding T25 or T18 domains alone served as a negative control. The right hand panel shows a schematic representation of the RapZ tetramer as seen in the crystal structure, and the individual domain interactions probed in this assay. (**B**) The β-strand comprising residues 179–182 is essential for self-interaction of the RapZ-CTD as revealed by BACTH. β-Galactosidase activities of strains producing variants of the RapZ-CTD fused to both, the T25- and T18-fragments of CyaA are shown in the left panel. The right panel shows the RapZ tetramer structure, with the CTD:CTD interaction probed in this assay highlighted in the dashed boxes. (**C**) BACTH analysis confirms the importance of residues Val29, Asn31 and Ser36 for self-interaction of the RapZ-NTD. β-Galactosidase activities of strains producing variants of the RapZ-NTD fused to both, the T25- and T18-fragments of CyaA are shown in the left panel. The right panel shows the RapZ tetramer structure, with the NTD:NTD interaction probed in this assay highlighted in the dashed boxes. (**D**) Mutation of residue Trp191 impairs the interaction between RapZ-NTD and CTD, but does not affect self-interaction of RapZ-CTD. β-Galactosidase activities of strains producing variants of the RapZ-NTD and RapZ-CTD fused to CyaA fragments are shown in the left panel. The right panel shows the RapZ tetramer structure, with the NTD:CTD interaction probed in this assay highlighted in the dashed boxes.

The crystal structures suggest that three distinct inter-protomer interfaces support the RapZ tetrameric assembly: the homotypic, symmetrical self-interactions between the N-terminal domain and the C-terminal domain, and two heterotypic interaction surfaces between the N- and C- terminal domains. To validate the proposed interactions we used a BACTH analysis which relies on reconstitution of the activity of a split adenylate cyclase (CyaA) through interaction of proteins fused to the split CyaA-T18 and -T25 domains (26). Indeed, high β-galactosidase activities reflecting high cAMP levels produced by reconstituted CyaA were measured for full-length RapZ confirming self-interaction (Figure 5A, column 5). Similarly high activities were measured when RapZ-NTDs and RapZ-CTDs were tested for the ability to form homo-multimers (Figure 5A, columns 1 and 4). Moreover, BACTH detected cross-interaction of the RapZ NTD and CTD, albeit the interaction appeared to be comparatively weaker (Figure 5A, columns 2 and 3). These data are in agreement with the various RapZ self-interactions predicted by the crystal structure.

The structure identifies numerous residues that are involved in forming the subunit interfaces. Self-interaction of the RapZ-CTD appears to be mediated by the beta-strand comprising residues 179–182 (YVFD). Accordingly, each of these residues was mutated to Ala (except for Val180, which was substituted by Gly) and individual mutants were examined in BACTH. Each mutation abolishes self-interaction of the CTD (aa 153–284) when present in both interacting monomers (Figure 5B), in agreement with the proposed dimer interface from the crystal structure. Similarly, self-interaction of the NTD appears to involve the short beta-strand comprising residues 27–30 (YCVD), the α-helix comprising residues 35–43 (VLLPDLART) and the adjacent linker (residues 31–34) (NLPV). Of these residues, the side-chains of Leu36, Asp39, Leu40 and Thr43 (all located in the helix) point to the dimer interface and could be involved in interaction. Additionally, the side chains of Tyr27 and Asn31 point into the dimer interface and might participate in interaction. To test their contribution to dimer formation, Val29 and Asn31 were mutated to Trp. Bulky substitutions here are predicted to disrupt the interface, and consistent with this hypothesis, the Val29→Trp and Asn31→Trp mutations both abolish self-interaction of the NTD (Figure 5C). In addition, a Leu36→Ser exchange also disrupted NTD self-interaction (Figure 5C). Trp191, which lies at the interface between the NTDs and CTDs of adjacent subunits, appears to be important in mediating tetramer stability as mutation of this residue to Ala impairs interaction of the CTD with the NTD without affecting CTD/CTD dimerization (Figure 5D). Cumulatively, the BACTH experiments provide compelling evidence in support of an oligomeric form of RapZ. To address what function this assembly serves, we explored the impact of oligomerization on RNA binding.

### Mutations impairing dimerization of the RapZ-CTD abolish protein function *in vivo*

**Figure 6.**
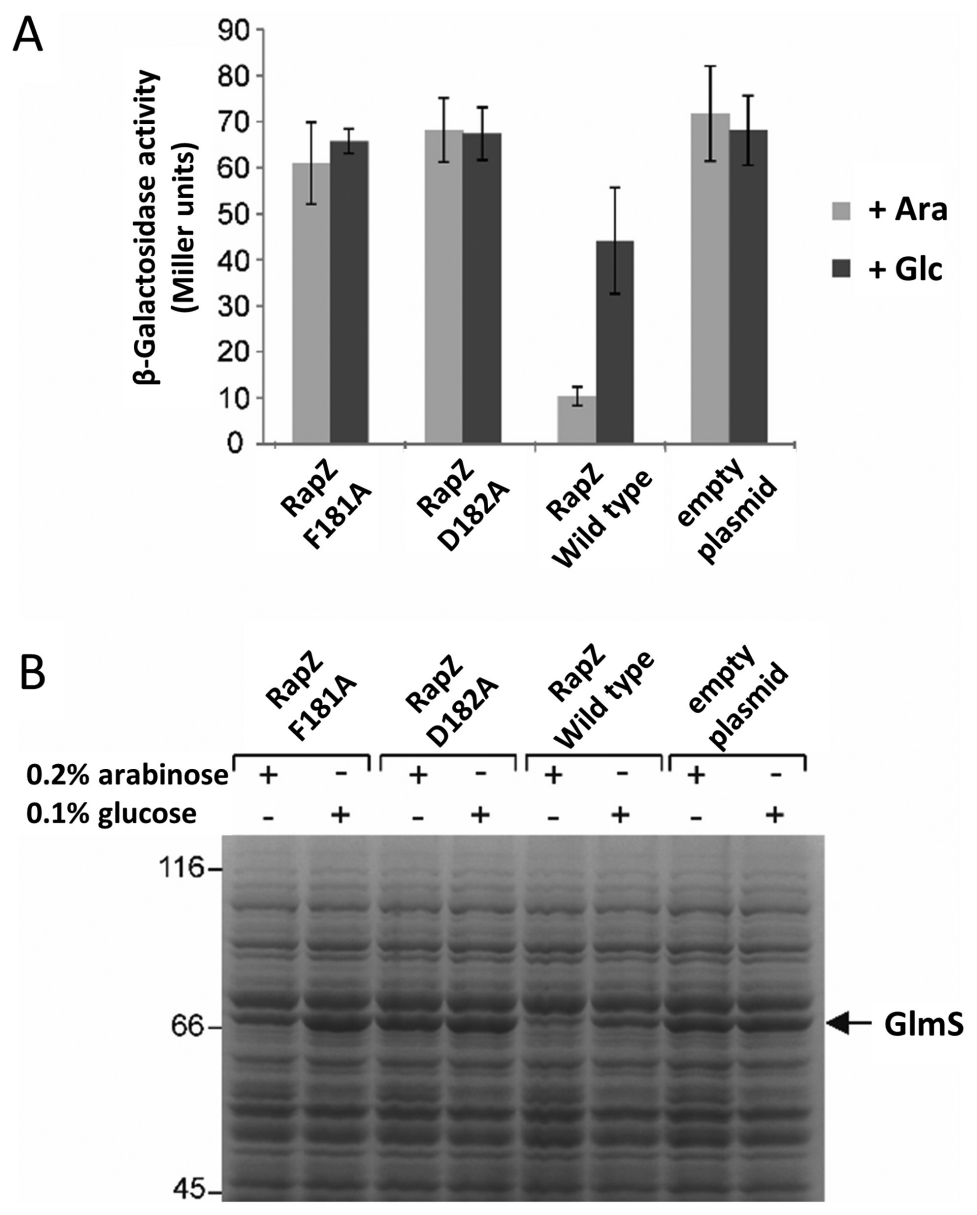
Oligomerization of RapZ is required for activity and RNA binding. (**A**) Complementation assay demonstrating that correct multimerization is essential for activity of RapZ *in vivo*. Plasmids encoding RapZ-F181A, RapZ-D182A or wild-type RapZ were introduced into strain Z28, which lacks the endogenous *rapZ* gene and carries an ectopic *glmS’-lacZ* reporter fusion in the *λattB* site on the chromosome. Cells carrying the empty expression vector pBAD33 (‘empty plasmid’) served as negative control. Arabinose (Ara) was added to induce and glucose (Glc) to repress expression of the *rapZ* alleles from the *P_Ara_* promoter. Cells were grown to exponential phase followed by assessment of β-galactosidase activity. (**B**) Total cell extracts from (A) were subjected to sodium dodecyl sulphate-polyacrylamide gel electrophoresis (SDS-PAGE) followed by Coomassie staining to directly assess the levels of GlmS, which becomes visible as a discrete band in *ΔrapZ* strains (5).

To assess the importance of inter-domain interactions for RapZ activity, we explored the impact of two CTD dimer-abolishing mutations, Phe181Ala and Asp182Ala, on activity of the full-length protein *in vivo*. A complementation assay was carried out in a *ΔrapZ* strain harbouring a *glmS’-lacZ* chromosomal reporter fusion. Wild-type *rapZ* and the two mutant derivatives were introduced on low copy plasmids where expression was under the control of an arabinose-inducible promoter. High *glmS* expression levels indicate an absence of GlmZ cleavage. As expected, both mutants did not complement a *rapZ* deletion, i.e. they are inactive (Figure 6A). With wild-type RapZ, *glmS* expression levels were reduced, but when the mutants were induced, *glmS* expression levels remained high, indicating that GlmZ cleavage did not occur. These data are supported by SDS-PAGE analysis of total cell extracts where a strong accumulation of GlmS protein is observed in the presence of the *rapZ* mutants (Figure 6B). As Phe181 and Asp182 are essential for dimerization of the CTD, this would indicate that correct oligomerization is essential for RapZ activity.

### The C-terminal domain of RapZ is sufficient to bind RNA

**Figure 7.**
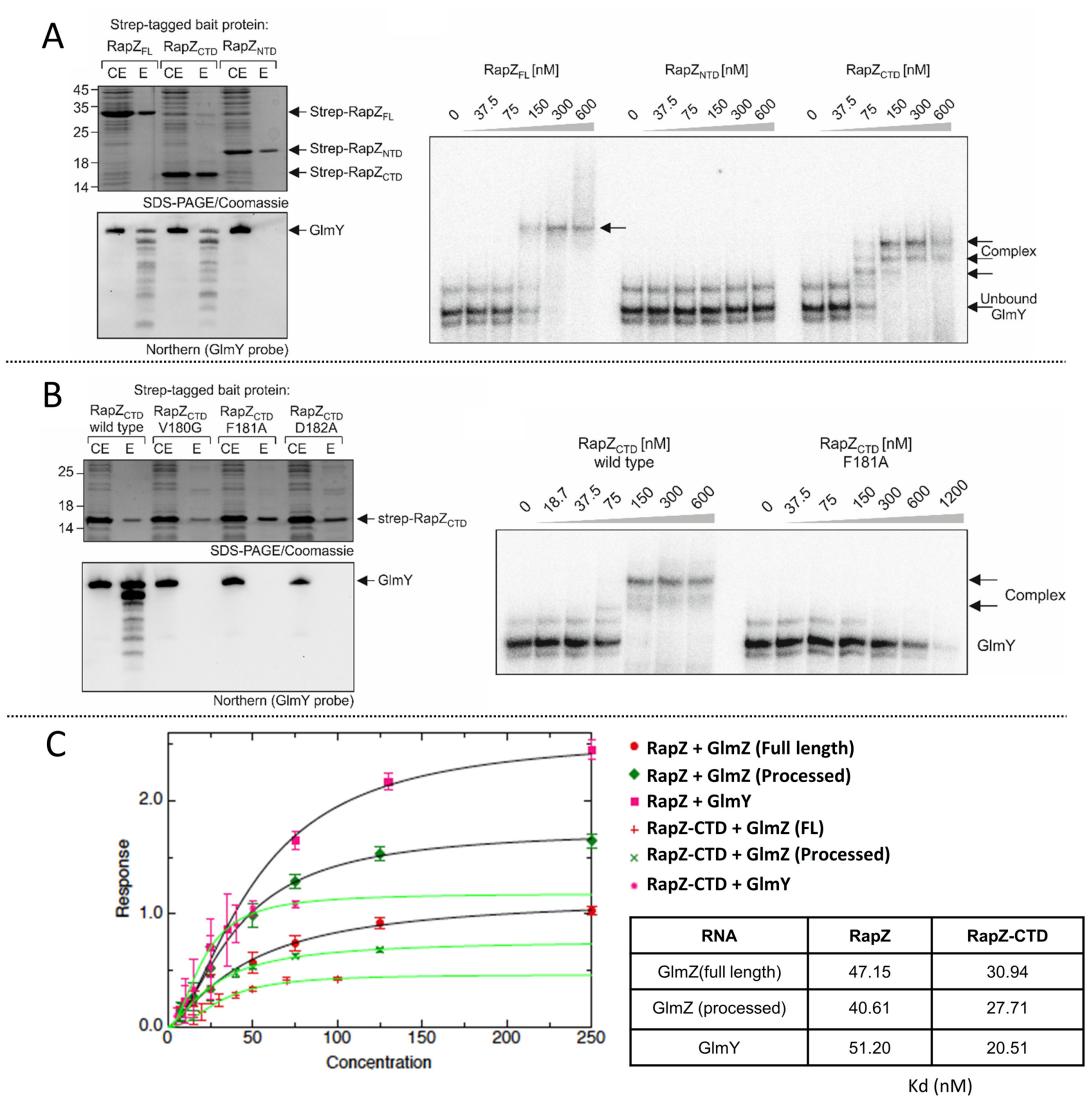
Assessment of the role of individual domains of RapZ in RNA binding. (**A**) RNA binding activity is associated with the RapZ-CTD but not with the NTD. Full-length Strep-RapZ, Strep-RapZ-CTD and Strep-RapZ-NTD were purified via StrepTactin affinity chromatography from strain Z903 lacking endogenous *rapZ*. Aliquots of the elution fractions (E) were analyzed by 15% SDS-PAGE/Coomassie staining (top) and in parallel used to extract RNA, which was subsequently analyzed by northern blotting using a probe specific for GlmY (bottom). The amounts of RNA loaded were adjusted to equal protein concentrations, as determined by the SDS-PAGE gel (top). In addition, the cell extracts (CE) of the various strains harvested prior to protein purification were analyzed alongside the elution fractions. Right: EMSA experiments using purified proteins and radio-labelled GlmY. (**B**) Wild-type Strep-RapZ-CTD or mutants V180G, F181A and D182A were purified by StrepTactin affinity chromatography from strain Z903 and analysed as in (A). Right: EMSA experiment showing that RapZ CTD harbouring the F181A mutation is unable to bind radiolabelled GlmY *in vitro*. (**C**) RapZ-CTD binds RNA targets with higher affinity than the full-length protein. Dissociation constants were calculated for full-length RapZ (1–284) or RapZ-CTD (154–284) by biolayer interferometry using an Octet Red 96 device. The measurements were performed by fitting the response from three independent experiments, and error bars represent standard deviations from the mean.

One possible explanation for the inactivity of the RapZ mutants could be that oligomerization is required for binding the small RNAs. EMSA and pull-down experiments using StrepTactin affinity chromatography indicate that the main RNA-binding activity of RapZ resides in its C-terminus (Figure 7A). The pulldown experiments demonstrate co-purification of GlmY with Strep-tagged full-length RapZ and RapZ-CTD, but not RapZ-NTD (Figure 7A, left). These observations are supported by EMSA experiments showing that RNA binding activity is exclusively associated with the CTD (Figure 7A, right). Next, we repeated the pull-down experiments but used RapZ-CTD variants carrying the dimer-abolishing mutations Val180Gly, Phe181Ala or Asp182Ala. Each of these mutations leads to complete loss of RNA binding *in vivo* (Figure 7B, left). Loss of RNA-binding activity is also supported *in vitro* by EMSA using the Phe181Ala mutant (Figure 7B, right).

We explored if there was a difference in the affinity of full-length RapZ and RapZ-CTD for their substrates utilizing BLI (Figure 7C). The full length and processed forms of GlmZ and processed GlmY were immobilized to streptavidin sensors via a 5′-terminal biotin and association rates were measured for RapZ and RapZ-CTD. Dissociation rates could not be measured as RapZ and RapZ-CTD do not fully dissociate from the sensors. As can be seen in Figure 7C, RapZ-CTD consistently has slightly higher affinity for its substrates than full-length RapZ by approximately a factor of 2. It may be the case that the N-terminal domain of RapZ plays an auto-inhibitory role in regulating RNA binding.

### Interaction between RapZ and RNase E requires the presence of both domains of RapZ

**Figure 8.**
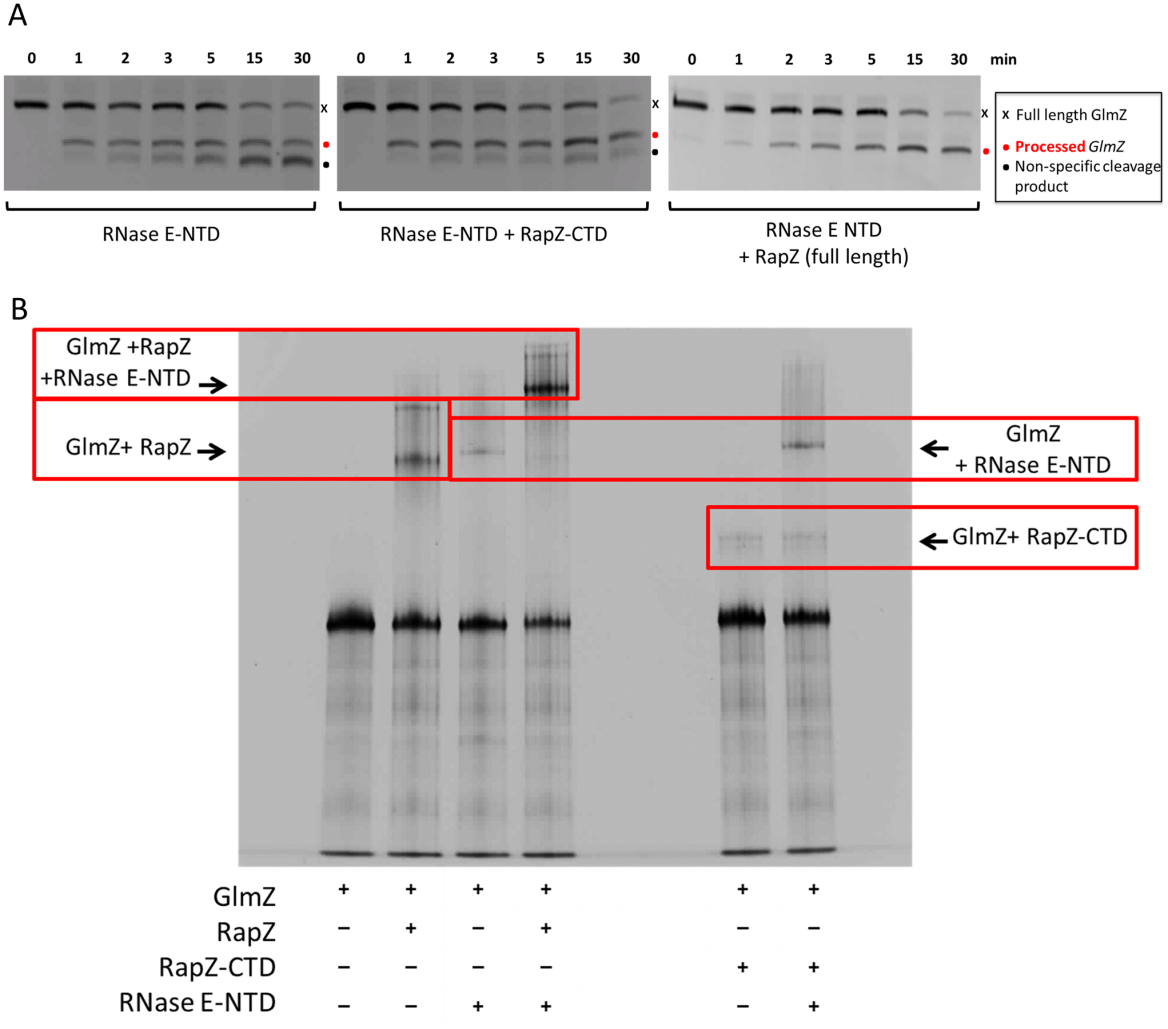
The role of RapZ domains in the interaction with RNase E. (**A**) GlmZ (20 nM) and RNase E NTD were mixed together in a 2:6 molar ratio and cleavage was allowed to proceed for the prescribed time at 30°C in the presence and absence of full length RapZ or the RapZ-CTD alone. The products of the degradation assay of GlmZ FL by catalytically active RNase E NTD (1–529) are identical irrespective of whether the C-terminal domain of RapZ is absent or present, whereas the presence of full length RapZ alters the processing pattern of GlmZ. (**B**) EMSA assays to probe the interaction between RNA, RNase E catalytic domain and RapZ domains. GlmZ, RapZ, RapZ-CTD and RNase E catalytic domain were mixed in equimolar ratios and incubated for 30 min at 30°C before being run on a native polyacrylamide gel and stained for RNA with SYBR Gold. Complexes are highlighted with red boxes.

Although RapZ-CTD is sufficient to bind the RNA targets of the full-length protein, this domain alone cannot efficiently mediate specific cleavage of GlmZ by the catalytic domain of RNase E. Cleavage assays of GlmZ using the RNase E catalytic domain alone and in the presence of either full-length RapZ or RapZ-CTD shown in Figure 8A reveal that RapZ-CTD is inefficient in mediating GlmZ processing. Additionally, EMSA data indicate that a ternary complex can form between RNase E catalytic domain, RapZ and GlmZ (Figure 8B). However, when using the RapZ-CTD, formation of the ternary complex cannot be observed. We are also unable to observe complex formation between the RapZ-CTD and RNase E catalytic domain *in vitro* following size exclusion chromatography (Supplementary Figure S3). Thus, the interaction of GlmZ with RapZ-CTD is insufficient to guide specific cleavage by RNase E, suggesting that the RapZ-NTD may be necessary for mediating contact with RNase E via a direct interaction on its surface or by facilitating the formation of a higher order assembly that RapZ-CTD alone cannot achieve.

## DISCUSSION

This study provides a structural rationale for understanding RapZ function in the control of amino-sugar metabolism. We have determined the crystal structures of full length RapZ in two different crystal forms at 3.4 Å and 3.24 Å resolution, and the isolated C-terminal RNA binding domain at 1.17 Å resolution. The structural data were used to guide *in vitro* and *in vivo* biochemical analyses of the mechanism of RapZ.

Considering first the RapZ-CTD, we have found that in isolation this proteolytically stable domain forms a homo-dimer both in solution and within our high resolution crystal structure. We have shown through a variety of binding assays and pull-down experiments that the RapZ-CTD is itself sufficient to bind RNA, and that dimer formation appears to be critical *in vivo* as dimer-abolishing mutations result in the loss of *glmS* regulation (Figure 6A). However, this domain alone is not sufficient to mediate the specific and efficient cleavage of GlmZ during *in vitro* degradation assays with the catalytic domain of RNase E, pointing towards a role for the N-terminal domain of RapZ in mediating the interaction with RNase E.

RapZ-CTD unexpectedly exhibits distant structural homology to the fructose-6-phosphate binding domain of PFK, suggesting a common evolutionary origin. Interestingly, recent studies addressing the global RNA interactomes in various organisms revealed that numerous metabolic enzymes have RNA-binding activity. Among them, enzymes involved in glycolysis and other carbohydrate metabolic pathways are in particular enriched (32–34). It is speculated that these enzymes possess moonlighting function, similar to aconitase and glyceraldehyde-3-phosphate dehydrogenase, which have roles in post-transcriptional gene regulation in bacteria as well as eukaryotes (35–38). This could also apply to PFK, which has been detected in the RNA interactomes of Arabidopsis and yeast and is also a component of the RNA-degradosome in Gram-positive bacteria (34,39). Perhaps RapZ uses an evolved RNA-binding domain originally derived from PFK to interact with sRNAs GlmY and GlmZ.

The RNA binding CTD of RapZ (and the structurally related domain from PFK) do not share homology with other known RNA interaction domains. Interestingly, many of the novel RNA-binding proteins identified in recent interactome studies are enriched in lysine and arginine residues (33,40). It is of note that 8 out of the 19 C-terminal residues in RapZ correspond to lysines and arginines and mutation of four of these residues abolishes RNA-binding activity (8). In our RapZ crystal structures, the far C-terminal residues are accessible on the surface of the molecule, further supporting the idea that this region is part of a novel RNA-binding motif.

During refinement of the high resolution crystal structure of RapZ-CTD there was clearly defined density for a non-water ligand. Considering the chemical components present in the crystallization solution we have modelled this ligand as malonate, and this molecule accounts for the ligand density excellently (Figure 3C). Although malonate is not considered to be a natural metabolite in *E. coli*, it does raise the possibility that the malonate binding site (formed predominantly by residues H190, T248, G249, H252 and R253) may represent a genuine binding site for an *E. coli* metabolite. Investigation of this potential metabolite binding pocket will form the basis of future studies.

In addition to the high resolution crystal structure of the RapZ-CTD, we have also solved the crystal structure of full length RapZ at 3.4 Å resolution. From this structure we were able to confirm previous predictions that the NTD of RapZ adopts a kinase like fold, with closest structural similarity found to an adenylate kinase-related protein from *S. solfataricus*. It is also interesting to note that there is electron density for a non-water ligand at the Walker A-box in this crystal structure, which we have modelled as a sulfate ion (Figure 4E).

The crystal structure and SEC-MALS experiments support the formation of a RapZ tetramer. Closer inspection of the RapZ crystal structure reveals that the tetramer is composed of two dimers of the RapZ-CTD as formed in the isolated CTD structure, and two dimers of the RapZ-NTD, brought together in a domain-swapped dimer-of-dimers. Through extensive mutational analyses we have provided evidence for the functional role of the tetrameric structure of RapZ in recognition of RNA and in the interaction with RNase E. Previously reported biophysical data indicate that RapZ is a trimer in solution (20), but our current evidence now strongly suggests that not only is RapZ in fact a tetramer in solution, but the inter-molecular contacts seen in our crystal structures are crucial for oligomerization in solution. Furthermore, RNA binding via the C-terminal RNA-binding domain requires formation of the dimer of the CTD of RapZ as seen in our crystal structures.

**Figure 9.**
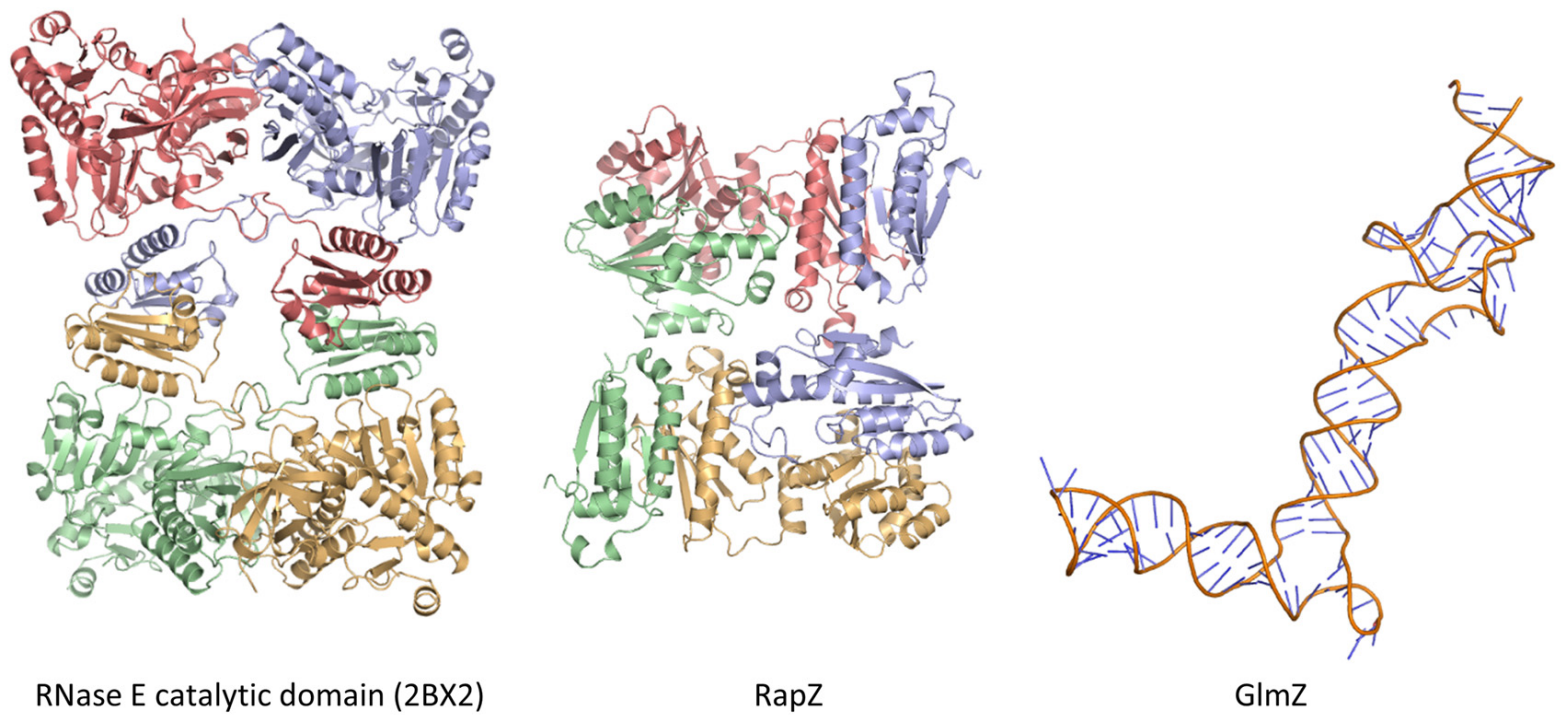
A structure gallery of components involved in RapZ-mediated regulation of bacterial amino-sugar metabolism. Left: the RNase E catalytic domain tetramer (PDB code: 2BX2) with the four protomers coloured red, blue, green and yellow. Middle: the RapZ tetramer with protomers coloured red, blue, green and yellow. Right: a molecular model of the post-cleavage GlmZ RNA (generated by SimRNAweb). The purpose of this model is to give an indication of the relative sizes of the ternary complex components, rather than to suggest the three-dimensional structure adopted by GlmZ. All models are shown in the same relative scale.

Our EMSA data indicate that a ternary complex can form between RNase E catalytic domain, RapZ and GlmZ (Figure 8). We envisage that a sandwiching complex might form at least transiently, in which GlmZ interacts with both RapZ and the catalytic domain of RNase E. For many substrates, RNase E activity can be enhanced by the presence of a 5′ monophosphate group, but the status of the 5′ end of GlmZ does not impact on its processing by RNase E (7). Instead, RNase E may sense secondary structural elements in GlmZ, similar to the mechanism involved in the 5′ bypass mechanism also utilized by RNase E (41). Recently obtained results indicate that the central stem loop of GlmZ is decisive for processing, while the actual cleavage site is dispensable (7). To give an indication of the relative size of the key components of this ternary complex and for the extent of surfaces available for interaction, a structural gallery is shown in Figure 9, with the RNase E catalytic domain tetramer (PDB code: 2BX2) (42), the RapZ tetramer and a hypothetical model of the post-cleavage GlmZ RNA generated by SimRNAweb (43). Similar to the RapZ protein, the RNase E catalytic domain is a homo-tetramer comprised of a dimer of dimers. The RNA binding surface of RapZ likely involves the CTDs presented in the tetramer, and GlmZ could be sandwiched between this surface and the RNase E in an encounter complex. Our crystallographic and biochemical analyses support a model in which the tetrameric, full length RapZ forms a complex with GlmZ that interacts with RNase E to guide specific cleavage of the sRNA, thereby down-regulating *glmS* activity.

## ACCESSION NUMBERS

The coordinates and structure factors have been deposited with the PDB, with accession codes 5O5O (full length RapZ, P32 space group), 5O5Q (full length RapZ, P3221 space group), 5O5S (RapZ-CTD).

## Supplementary Material

Supplementary Data
