# Supplementary material for: Structural insights into RapZ-mediated regulation of bacterial amino-sugar metabolism
Source: Nucleic Acids Res. 2017 Sep 5;45(18):10845–60. doi: 10.1093/nar/gkx732 (PMC5737377; doi:10.1093/nar/gkx732)
Supplement: Supplementary Data [file gkx732_supp.pdf]

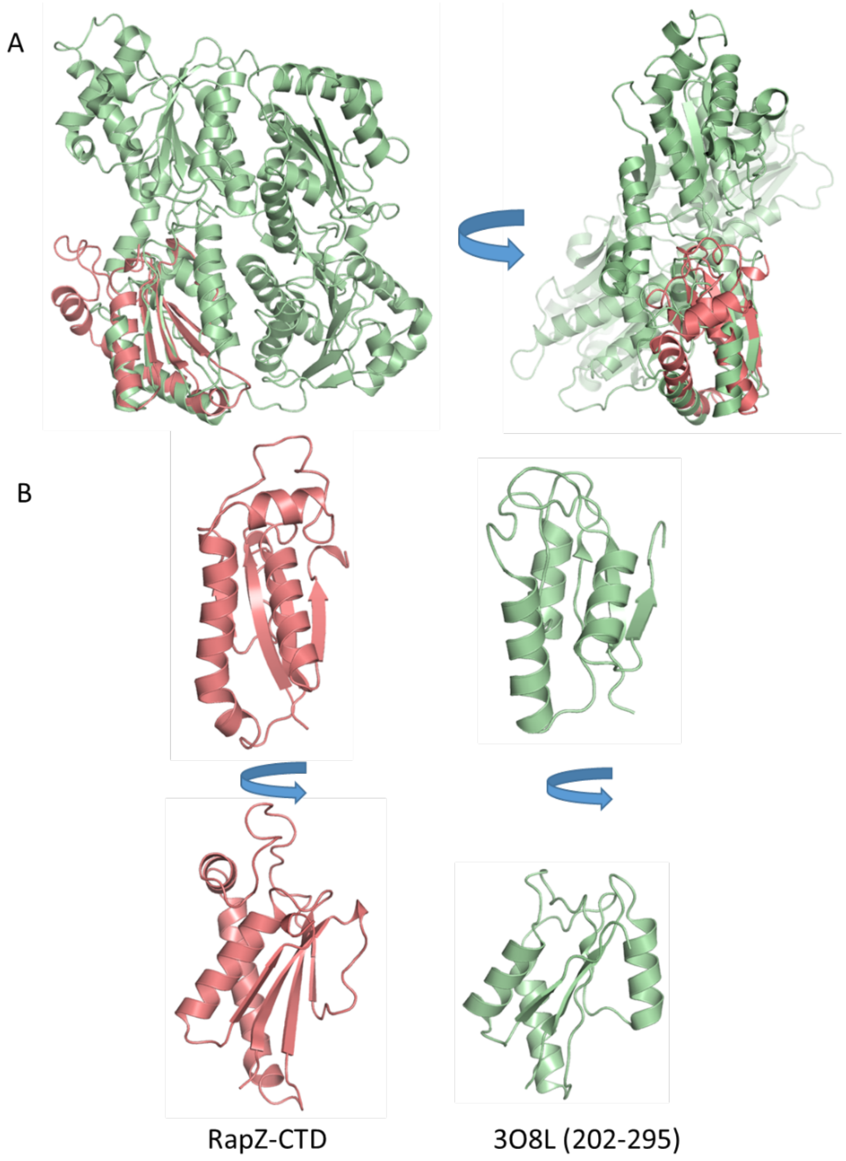

Supplementary Figure 1. Comparison of RapZ-CTD and Phosphofructokinase. A. The top scoring structural match to RapZ-CTD as determined by the DALI webserver is rabbit skeletal muscle phosphofructokinase (PDB 3O8L). A protomer of RapZ-CTD is shown as red cartoon representation superimposed on the equivalent region within a protomer of PFK (green cartoon). B. Two views of a RapZ-CTD protomer are shown (red cartoon, left) in the same orientation as the region of PFK matching the RapZ-CTD (residues 202-295, green cartoon, right).

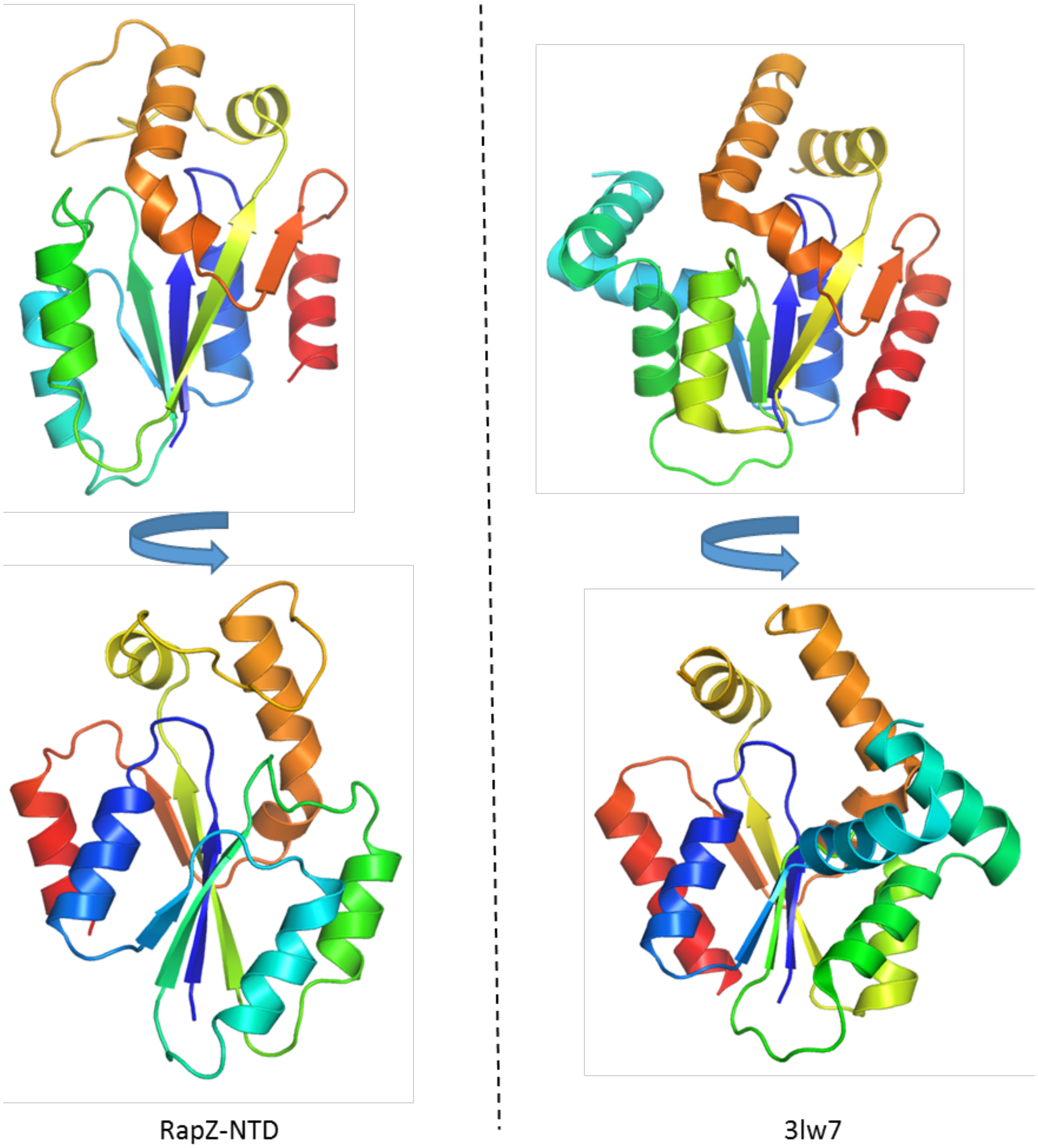

Supplementary Figure 2. Comparison of RapZ-NTD and adenylate-kinase enzymes. The top scoring structural match to RapZ-NTD as determined by the DALI webserver is an adenylate kinase-related protein from *Sulfolobus solfataricus* (PDB 3LW7). Two views of a RapZ-NTD protomer are shown on the left as cartoon representation coloured in blue to red rainbow from N to C terminus. Right: two views of a protomer of the *S. solfataricus* adenylate kinase in the same orientation as the RapZ-NTD, coloured in blue to red rainbow from N to C terminus.

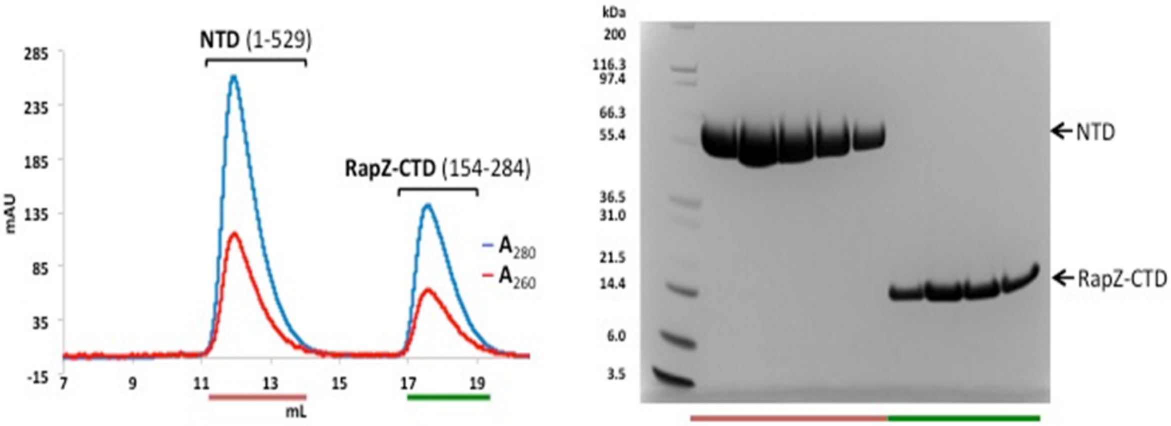

Supplementary Figure 3. The RapZ-CTD and RNase E-NTD do not directly interact. Left: gel filtration chromatogram of a mixture of RNase E-NTD and RapZ-CTD. Right: SDS-PAGE analysis of the underlined "red" and "green" peaks, indicating that the two proteins do not co-elute from the gel fitration column.

SUPPLEMENTARY MATERIALS AND METHODS

For construction of plasmids strain Xl1-blue was used. Site-directed mutagenesis was performed using the combined chain reaction (CCR) protocol (Bi & Stambrook, 1998). Briefly, 5'-phosphorylated oligonucleotides carrying the desired mutations were incorporated by thermostable ampligase into PCR fragments during amplification. Two hybrid constructs created in this study encode fusions of full-length or truncated RapZ variants to T25 or T18 domains of *B. pertussis* adenylate cyclase. To construct plasmids which carry fusions of RapZ NTD (aa 1-152) to T25 or T18 domain, inserts were PCR-amplified from full-length *rapZ* using BG637/BG1222 primers, digested with XbaI and KpnI and cloned into pKT25 (creating pSD9) or pUT18C (creating pSD11) plasmids. Similarly, for constructs which encode RapZ CTD (aa 153-284) fused to T25 or T18 domain, PCR fragments were amplified from full-length *rapZ* utilizing BG1223/BG639 primers and inserted between XbaI/KpnI sites in pKT25 (pSD10) or pUT18C (pSD12) plasmids. RapZ NTD mutants harboring V29W and N31W exchanges were introduced by CCR using phosphorylated mutagenesis primers BG1396 (V29W, pSD53) and BG1397 (N31W, pSD54). Forward and reverse primers as well as subsequent cloning procedure were

identical to those described for pSD9. Plasmid pSD57 is also isogenic to pSD9, but carries a L36S mutation in the *rapZ* gene. It was generated as described for plasmid pSD9, but plasmid “rapZ 1.1” was used as template for PCR. Plasmid “rapZ 1.1” was recovered in a genetic screen and will be described elsewhere. To establish V29W, N31W and L36S exchanges in RapZ NTD fused to T18, inserts were excised via XbaI/KpnI from pSD53, pSD54 and pSD57 and cloned into pUT18C thereby generating pSD64 (V29W), pSD65 (N31W) and pSD80 (L36S), respectively. Mutations Y179A, F181A and D182 were initially introduced into full-length *rapZ* via CCR, using mutagenesis primers BG1388, BG1367 and BG1213, respectively, and BG637/BG639 as flanking primers. The resulting CCR fragments were digested with XbaI/KpnI and ligated to pKT25, giving rise to plasmids pSD46 (Y179A), pBGG455 (F181A) and pBGG432 (D182A). The sequences encoding the RapZ-CTD were amplified from these plasmids using oligos BG1223/BG639 and inserted between the XbaI/KpnI sites of either pKT25 creating pSD48 (Y179A), pSD30 (F181A) and pSD32 (D182A), or of pUT18C thus generating pSD60 (Y179A), pSD31 (F181A) and pSD33 (D182A). Mutations V180G and W191A were introduced directly in the context of *rapZ-CTD* via CCR using primers BG1223/BG639 and mutagenesis primers BG1461 (V180G) and BG1395 (W191A). Obtained fragments were cloned between the XbaI/KpnI sites on either plasmid pKT25 creating pSD97 (V180G) and pSD52 (W191A) or on plasmid pUT18C giving rise to pSD96 (V180G).

In order to generate complementation constructs expressing *rapZ* -F181A or *rapZ*-D182A from the arabinose-inducible P_{Ara} promoter, CCRs were performed using outward primers BG1049/BG397 and the mutagenesis primers BG1367 and BG1213, respectively. The resulting CCR fragments were inserted between the SacI/XbaI sites on plasmid pBAD33 generating plasmids pBGG459 and pBGG436.

For construction of a plasmid encoding the Strep-tagged RapZ NTD, a fragment was amplified from full-length *rapZ* using BG1015/BG1358 primers, and ligated between the NheI/XbaI sites on plasmid pBGG237 thus creating pSD25. Using primers BG1340/BG397 plasmid pSD24 coding for Strep-RapZ CTD was created in a similar manner. Plasmids pSD98, pSD50, and pSD99 are isogenic to pSD24 but harbor mutations V180G, F181A and D182A, respectively. The *rapZ*-CTD fragments carrying mutations F181A (pSD50) and D182A (pSD99) were amplified using primers BG1340/BG397 and plasmids pBGG455 and pBGG432 as templates, respectively, and subsequently the PCR fragments were ligated with the NheI/XbaI-digested plasmid pBGG237. Plasmid pSD98 is isogenic but carries mutation V180G in *rapZ*. It was obtained by ligation of a CCR fragment, which was generated using primers BG1340/BG397 and mutagenesis primer BG1461, between the NheI/XbaI-sites on plasmid pBGG237.

Table S1. Strains and plasmids used in this study

Name	Relevant structure/genotype	Reference or construction
Strains		
BTH101	F^{-}, *cyaA-99, araD139, galE15, galK16, rpsL1 (Str^{R}), hsdR2, mcrA1, mcrB1*	(Karimova *et al.*, 1998)
R1279	CSH50 Δ(*pho-bgl*)201 Δ(*lac-pro*) *ara thi*	(Schnetz *et al.*, 1996)
TM529	W3110 mlc *rne598-FLAG-cat*	(Morita *et al.*, 2004)
Xl1 blue	*recA1 endA1 gyrA96 thi-1 hsdR17 supE44 relA1 lac [F' proAB lacI^{q} lacZ*Δ*M15* Tn*10 (Tet^{r})]*	Laboratory stock
Z28	as R1279, but Δ*rapZ, attB::*[*aadA, glmS*-5'::*lacZ*], *str^{R}* , *F'*(*pro^{+}*)	(Kalamorz *et al.*, 2007)
Z37	as R1279, but Δ*rapZ*	(Kalamorz *et al.*, 2007)
Z864	as R1279, but Δ*rapZ,* Δ*glmY,* Δ*glmZ*	(Göpel *et al.*, 2016)
Z903	as Z37, but *rne598-FLAG-cat*	T4GT7 (TM529) → Z37, this work
Plasmids		
pBAD33	*P$_{Ara}$*, MCS 2, *cat*, ori p15A	(Guzman *et al.*, 1995)
pBGG61	*rapZ* (-17 to +855) under *P$_{Ara}$*-control in pBAD33	(Göpel *et al.*, 2013)
pBGG164	*lacI^{q}*, *P$_{tac}$-streptag-rapZ*, *rrnBT1/T2*, *bla*, ori ColEI	(Lüttmann *et al.*, 2012)
pBGG237	*lacI^{q}*, *P$_{tac}$-streptag*-MCS, *rrnBT1/T2*, *bla*, ori ColEI	(Lüttmann *et al.*, 2012)
pBGG348	encodes T25-RapZ fusion in pKT25	(Göpel *et al.*, 2013)
pBGG349	encodes T18-RapZ fusion in pUT18C	(Göpel *et al.*, 2013)
pBGG432	as pBGG348, but *rapZ* with D182A mutation	this work
pBGG436	as pBGG61, but *rapZ* with D182A mutation	this work
pBGG455	as pBGG348, but *rapZ* with F181A mutation	this work
pBGG459	as pBGG61, but *rapZ* with F181A mutation	this work
pFDX4324	*rapZ* expressed from constitutive *P$_{tac}$, cat, ori pSC101*	(Kalamorz *et al.*, 2007)
pKT25	*P$_{lac}$::cyaA-T25* (aa 1-224), MCS, *neo*, ori p15A	(Karimova *et al.*, 1998)
pSD9	encodes T25-RapZ (aa 1-152) fusion in pKT25	this work
pSD10	encodes T25-RapZ (aa 153-284) fusion in pKT25	this work
pSD11	encodes T18-RapZ (aa 1-152) fusion in pUT18C	this work
pSD12	encodes T18-RapZ (aa 153-284) fusion in pUT18C	this work
pSD24	*strep-rapZ* (aa 153-284) under *P$_{tac}$* promoter in pBGG237	this work
pSD25	*strep-rapZ* (aa 1-152) under *P$_{tac}$* promoter in pBGG237	this work
pSD30	as pSD10, but *rapZ* with F181A mutation	this work
pSD31	as pSD12, but *rapZ* with F181A mutation	this work
pSD32	as pSD10, but *rapZ* with D182A mutation	this work
pSD33	as pSD12, but *rapZ* with D182A mutation	this work
pSD46	as pBGG348, but *rapZ* with Y179A mutation	this work
pSD48	as pSD10, but *rapZ* with Y179A mutation	this work
pSD50	as pSD24, but *rapZ* with F181A mutation	this work
pSD52	as pSD10, but *rapZ* with W191A mutation	this work
pSD53	as pSD9, but *rapZ* with V29W mutation	this work
pSD54	as pSD9, but *rapZ* with N31W mutation	this work
pSD57	as pSD9, but *rapZ* with L36S mutation	this work
pSD60	as pSD12, but *rapZ* with Y179A mutation	this work
pSD64	as pSD11, but *rapZ* with V29W mutation	this work
pSD65	as pSD11, but *rapZ* with N31W mutation	this work
pSD80	as pSD11, but *rapZ* with L36S mutation	this work
pSD96	as pSD12, but *rapZ* with V180G mutation	this work
pSD97	as pSD10, but *rapZ* with V180G mutation	this work
pSD98	as pSD24, but *rapZ* with V180G mutation	this work
pSD99	as pSD24, but *rapZ* with D182A mutation	this work
pUT18C	*P$_{lac}$ cyaA-T18* (aa 225-399), MCS, *bla*, ori ColEI	(Karimova *et al.*, 1998)

Table S2. Oligonucleotides used in this study

Primer	Sequence	Res. Sites	Position
BG397	TGGCTGCAGTCTAGATTATCATGGTTTACGTTTTTCCAGCG	PstI, XbaI	*rapZ* +855 to +833
BG637	GCGTCTAGA**G**ATGGTACTGATGATCGTCAGCG	XbaI	*rapZ* +1 to +22
BG639	CGCGGTACCTCATGGTTTACGTTTTTCCAGCG	KpnI	*rapZ* +855 to +833
BG1015	GGCTGCTAGCATGGTACTGATGATCGTCAGCG	NheI	*rapZ* +1 to +22
BG1049	GGCGAGCTCGTGAGGAGAAACAGTACATGGTACTGATGATCGTCAGCG	SacI	*rapZ* -17 to +22
BG1213	[P]-CCTATCGATGCAGATTACGTCTTTG**C**CGTGCGCTTCTTGCCGAACCCG		*rapZ* +520 to +567
BG1222	CGCGGTACC**TTA**CAGACGGGTACGCAGCATTTC	KpnI	*rapZ* +456 to +436
BG1223	GCGTCTAGA**G**CTGGGTAAACGTGAACGCG	XbaI	*rapZ* +457 to +475
BG1340	GGCTGCTAGCCTGGGTAAACGTGAACGCGAAC	NheI	*rapZ* +457 to +478
BG1358	GCGTCTAGA**TTA**CAGACGGGTACGCAGCATTT		*rapZ* +456 to +437
BG1367	[P]-GATGCAGATTACGTCGCTGACGTGCGCTTC		*rapZ* +526 to +555
BG1388	[P]-CCTATCGATGCAGAT**GC**CGTCTTTGACGTGC		*rapZ* +520 to +550
BG1395	[P]-CCGAACCCGCAC**GC**GGATCCGAAACTGC		*rapZ* +559 to +586
BG1396	[P]-GATATGGGTTTTTACTGC**TG**GGATAACCTTCCCG		*rapZ* +67 to +100
BG1397	[P]-GTTTTTACTGCGTGGAT**TGG**CTTCCCGTAGTGTTG		*rapZ* +74 to +108
BG1461	[P]-CGATGCAGATTACG**G**CTTTGACGTGCGC		*rapZ* +525 to +552
rapZ_F	TACTTCCAATCCAATGCGATGTGGTCGCATCCGCAGTTTGAAAAG GAGAACCTGTACTTCCAATCCATGGTACTGATGATC		
rapZ_R	TTATCCACTTCCAATGTTATGGTTTACGTTTTTC		
rapZ_CTD_F	TACTTCCAATCCAATGCGATGTGGTCGCATCCGCAGTTTGAAAAG GAGAACCTGTACTTCCAATCCGGTAAACGTGAACGC		

Restriction sites are underlined; [P] denotes 5´-phosphorylation of oligonucleotide. Deviations from wild type sequence are in bold, and dashed line corresponds to T7 promoter sequence. Position relates to the first nucleotide of the respective gene.

Supplementary References

1. Bi, W. and Stambrook, P.J. (1998) Site-directed mutagenesis by combined chain reaction. *Anal. Biochem.*, **256**, 137-140.
2. Karimova, G., Pidoux, J., Ullmann, A. and Ladant, D. (1998) A bacterial two-hybrid system based on a reconstituted signal transduction pathway. *Proc. Natl. Acad. Sci. U S A*, **95**, 5752-5756.
3. Schnetz, K., Stülke, J., Gertz, S., Krüger, S., Krieg, M., Hecker, M. and Rak, B. (1996) LicT, a *Bacillus subtilis* transcriptional antiterminator protein of the BglG family. *J. Bacteriol.*, **178**, 1971-1979.
4. Morita, T., Kawamoto, H., Mizota, T., Inada, T. and Aiba, H. (2004) Enolase in the RNA degradosome plays a crucial role in the rapid decay of glucose transporter mRNA in the response to phosphosugar stress in *Escherichia coli*. *Mol. Microbiol.*, **54**, 1063-1075.

5. Kalamorz, F., Reichenbach, B., März, W., Rak, B. and Görke, B. (2007) Feedback control of glucosamine-6-phosphate synthase GlmS expression depends on the small RNA GlmZ and involves the novel protein YhbJ in *Escherichia coli*. *Mol. Microbiol.*, **65**, 1518-1533.
6. Göpel, Y., Khan, M.A. and Görke, B. (2016) Domain swapping between homologous bacterial small RNAs dissects processing and Hfq binding determinants and uncovers an aptamer for conditional RNase E cleavage. *Nucleic Acids Res.*, **44**, 824-837.
7. Guzman, L.M., Belin, D., Carson, M.J. and Beckwith, J. (1995) Tight regulation, modulation, and high-level expression by vectors containing the arabinose P_{BAD} promoter. *J. Bacteriol.*, **177**, 4121-4130.
8. Göpel, Y., Papenfort, K., Reichenbach, B., Vogel, J. and Görke, B. (2013) Targeted decay of a regulatory small RNA by an adaptor protein for RNase E and counteraction by an anti-adaptor RNA. *Genes Dev.*, **27**, 552-564.
9. Lüttmann, D., Göpel, Y. and Görke, B. (2012) The phosphotransferase protein EIIA(Ntr) modulates the phosphate starvation response through interaction with histidine kinase PhoR in *Escherichia coli*. *Mol. Microbiol.*, **86**, 96-110.
